# Trace Elements Induce Predominance among Methanogenic Activity in Anaerobic Digestion

**DOI:** 10.3389/fmicb.2016.02034

**Published:** 2016-12-16

**Authors:** Babett Wintsche, Karin Glaser, Heike Sträuber, Florian Centler, Jan Liebetrau, Hauke Harms, Sabine Kleinsteuber

**Affiliations:** ^1^Department of Environmental Microbiology, Helmholtz Centre for Environmental Research – UFZLeipzig, Germany; ^2^Department of Applied Ecology and Phycology, University of RostockRostock, Germany; ^3^Department of Biochemical Conversion, Deutsches Biomasseforschungszentrum – DBFZLeipzig, Germany; ^4^German Centre for Integrative Biodiversity Research (iDiv)Leipzig, Germany

**Keywords:** biogas reactor, methanogenesis, *mcr*A, *Methanosarcina*, *Methanoculleus*, amplicon sequencing, T-RFLP

## Abstract

Trace elements (TE) play an essential role in all organisms due to their functions in enzyme complexes. In anaerobic digesters, control, and supplementation of TEs lead to stable and more efficient methane production processes while TE deficits cause process imbalances. However, the underlying metabolic mechanisms and the adaptation of the affected microbial communities to such deficits are not yet fully understood. Here, we investigated the microbial community dynamics and resulting process changes induced by TE deprivation. Two identical lab-scale continuous stirred tank reactors fed with distiller’s grains and supplemented with TEs (cobalt, molybdenum, nickel, tungsten) and a commercial iron additive were operated in parallel. After 72 weeks of identical operation, the feeding regime of one reactor was changed by omitting TE supplements and reducing the amount of iron additive. Both reactors were operated for further 21 weeks. Various process parameters (biogas production and composition, total solids and volatile solids, TE concentration, volatile fatty acids, total ammonium nitrogen, total organic acids/alkalinity ratio, and pH) and the composition and activity of the microbial communities were monitored over the total experimental time. While the methane yield remained stable, the concentrations of hydrogen sulfide, total ammonia nitrogen, and acetate increased in the TE-depleted reactor compared to the well-supplied control reactor. *Methanosarcina* and *Methanoculleus* dominated the methanogenic communities in both reactors. However, the activity ratio of these two genera was shown to depend on TE supplementation explainable by different TE requirements of their energy conservation systems. *Methanosarcina* dominated the well-supplied anaerobic digester, pointing to acetoclastic methanogenesis as the dominant methanogenic pathway. Under TE deprivation, *Methanoculleus* and thus hydrogenotrophic methanogenesis was favored although *Methanosarcina* was not overgrown by *Methanoculleus*. Multivariate statistics revealed that the decline of nickel, cobalt, molybdenum, tungsten, and manganese most strongly influenced the balance of *mcrA* transcripts from both genera. Hydrogenotrophic methanogens seem to be favored under nickel- and cobalt-deficient conditions as their metabolism requires less nickel-dependent enzymes and corrinoid cofactors than the acetoclastic and methylotrophic pathways. Thus, TE supply is critical to sustain the activity of the versatile high-performance methanogen *Methanosarcina*.

## Introduction

Anaerobic digestion (AD) of organic waste and residues is an important component of renewable energy systems, advanced biorefineries, and sustainable waste management strategies. The biogas produced can be used to generate electricity and heat or can be upgraded to biomethane which is used as vehicle fuel or injected into the gas grid.

Anaerobic digestion is a complex multi-stage process relying on the activity of highly diverse microbial communities. Next to the macronutrients carbon, nitrogen, phosphorus and sulfur, trace elements (TE) are crucial for an effective biogas process due to the microbial demand for TE in the anaerobic environment (Demirel and Scherer, 2011). These demands are as diverse as the involved microorganisms and their functions. Many industrial biogas reactors in Germany are operated with energy crops such as maize silage as substrate. For maize silage it is known that its content of macro- and microelements is insufficient for the demands of anaerobic microorganisms. For instance, Lebuhn et al. (2008) showed that long-term mono-digestion of maize silage led to acidification and process failure even at low organic loading rates but the process recovered after TE supplementation. The authors concluded that cobalt was the most limiting element. In another study, both cobalt and nickel limitations caused process instability and decreased biogas production during AD of a model substrate for maize silage (Pobeheim et al., 2011). Stability of AD processes and efficient methane production are also impaired by deficiencies of other TE, for example molybdenum, tungsten or selenium (Plugge et al., 2009; Worm et al., 2009; Banks et al., 2012; Munk and Lebuhn, 2014).

To avoid a possible undersupply of TE, commercial TE supplements are added to biogas reactors based on the operator’s experience (Lemmer et al., 2010; Schmidt, 2011; Lindorfer et al., 2012; Schmidt et al., 2013; Evranos and Demirel, 2015). Correct dosing of TE supplements is very important, since undersupply can cause process instability or low methane yield, whereas overdosage may have toxic effects on the microorganisms and impairs the compliance of the digestate with the requirements for fertilizer (Thanh et al., 2016). To achieve an optimal TE supplementation and raise the efficiency of the AD process, detailed knowledge about essential and beneficial TE and their role in AD would be instrumental.

TE play integral roles in enzymatic complexes, for example as central ions conferring catalytic functions. Microorganisms involved in AD have specific TE requirements. Molybdenum, tungsten and selenium are essential TE for syntrophic bacteria (Vorholt and Thauer, 2002; Plugge et al., 2009; Worm et al., 2009) involved in the acetogenesis, i.e., converting volatile fatty acids (VFA) and alcohols to precursors of methanogenesis. The methane producing steps of AD also depend on several TE. For instance, the acetyl-CoA decarbonylase/synthase complex, the cofactor F430 and different hydrogenases – all key enzymes of methanogenic archaea – incorporate nickel (Deppenmeier et al., 1999; Thauer et al., 2008). Further essential TE in methanogenesis include cobalt and molybdenum or tungsten, which are the central ions of *S*-methyl-tetrahydrosarcinapterine and 5-methyl-tetrahydromethanopterine or the formylmethanofuran dehydrogenase, respectively (Vorholt and Thauer, 2002).

The methane-producing step in the AD process is exclusively performed by methanogenic archaea. Methanogenic communities are characterized by a lower diversity and lower functional redundancy than the highly diverse bacterial communities. Consequently, process conditions, which are adverse for methanogens can compromise process stability (Demirel, 2014). Methanogens are metabolically versatile and produce biogas by acetoclastic, methylotrophic or hydrogenotrophic methanogenesis (Costa and Leigh, 2014). During acetoclastic methanogenesis, methane is directly produced from acetate. All acetoclastic methanogens belong to the order *Methanosarcinales*. Particularly species of the genus *Methanosarcina* are considered as robust and effective methane producers occurring in high performance AD processes (Conklin et al., 2006; De Vrieze et al., 2012). They show high growth rates on diverse substrates (acetate, methanol, methylamines, or CO_2_ and H_2_) and are tolerant to fluctuating pH values and high ammonia concentrations (Liu and Whitman, 2008; Schnürer and Nordberg, 2008). *Methanosarcina* sp. are further capable of conducting hydrogenotrophic methanogenesis meaning that they can act as syntrophic partners of VFA degraders (Hao et al., 2011; Shimada et al., 2011; Karlsson et al., 2012). Thus, members of the genus *Methanosarcina* are usually regarded as ‘robust workhorses’ of AD (Willy Verstraete in his plenary lecture at the 13th World Congress on Anaerobic Digestion, Santiago de Compostela, June 25, 2013).

In the course of hydrogenotrophic methanogenesis, methane is formed from CO_2_ and H_2_ or formate. These substrates are products of the bacterial degradation processes acidogenesis and acetogenesis (Demirel and Scherer, 2008). Concentrations of formate and H_2_ in the system determine the activity of syntrophic bacteria degrading VFA, alcohols, etc., because these processes become thermodynamically feasible only when methanogenesis maintains low concentrations of formate and H_2_ (McInerney et al., 2009; Sieber et al., 2014). Hence, the presence of hydrogenotrophic methanogens is essential to keep the AD process running. However, the abundance and activity of hydrogenotrophic methanogens as well as their share of the total methane production depend on the process conditions (Karakashev et al., 2005).

The impact of different TE on the AD process and reactor performance has been addressed by numerous studies. Furthermore, biochemical backgrounds of the requirements of several TE have been studied closely in pure cultures. However, little is known about how TE deficiencies in AD influence the microbial community and which metabolic pathways are impacted in a way that community changes and process instabilities occur.

The aim of the present study was to investigate the effect of a slowly increasing TE deficit on reactor performance and the microbial communities in a semi-continuous AD process. After parallel operation of two lab-scale reactors, which were well supplied with TE, the TE supply of one reactor was stopped. Besides various process parameters, the dynamics of bacterial and methanogenic communities were monitored by T-RFLP (terminal restriction fragment length polymorphism) fingerprinting and sequencing of phylogenetic marker genes and their transcripts. The community dynamics were correlated to process parameters and TE concentrations to unravel the role of TE in AD along with their impact on bacterial and methanogenic communities.

## Materials and Methods

### Lab-Scale Biogas Reactors and Operation Conditions

Two identical lab-scale continuous stirred tank reactors designated R1 and R2 (total volume: 15 L; working volume: 10 L) were operated for 93 weeks with continuous stirring at 50 rpm using an anchor agitator propelled by an overhead stirrer RZR 2102 control (Heidolph, Germany). The temperature was kept constant at 37°C (±1 K) controlled by a water bath. A construction scheme of the lab-scale reactors used was given by Schmidt (2011). The inoculum was obtained from a running lab-scale biogas reactor operated with dried distiller’s grains with solubles (DDGS; CropEnergies AG, Germany). A mixture of 53.1 g DDGS, 2.57 g of a commercial iron additive for sulfide precipitation, and 2 mL of a TE stock solution containing cobalt, nickel, molybdenum and tungsten was dissolved in 345 mL water and fed daily as described by Schmidt et al. (2013) who found that efficient AD of DDGS requires supplementation of these TE. The TE mixture was composed of 2.13 g L^-1^ Ni(II)Cl_2_ × 6H_2_O (AppliChem, Germany), 0.531 g L^-1^ Co(II)Cl_2_ × 6H_2_O (AppliChem, Germany), 0.332 g L^-1^ NaMoO_4_ × 2H_2_O (Merck KGaA, Germany), and 4.268 g L^-1^ (NH_4_)_4_H_2_W_12_O_40_ × H_2_O (Sigma-Aldrich, USA). All TE salts were analytically pure.

The reactors were operated at an organic loading rate of 5 g_V S_ L^-1^ d^-1^ (VS: volatile solids) resulting in a hydraulic retention time of 25 days. Both reactors were operated in parallel for 72 weeks before starting the experimental period, during which R2 was subjected to TE decline by omitting the TE solution and reducing the supply of iron additive from 2.57 to 0.86 g per day.

### Analysis of Process Parameters and Analytical Techniques

Gas production, gas composition, and pH value were measured daily. The biogas volume was measured using drum-type gas meters TG 05 (Ritter, Germany) and normalized to dry gas at standard pressure (101.325 kPa) and standard temperature (273.15 K). The biogas composition (CH_4_, CO_2_, H_2_, H_2_S, and O_2_) was analyzed by an AWIFLEX gas analyzer (AWITE Bioenergie, Germany).

The total organic acids/alkalinity ratio and VFA concentrations were measured twice per week. Total ammonium nitrogen (TAN) concentrations were generally determined twice per week with a few exceptions when only one sample per week was measured. The total organic acids/alkalinity ratio and VFA concentrations were measured in triplicates and TAN concentrations in single measurements as described by Ziganshin et al. (2011). Total solids and volatile solids contents of substrates and digestates were determined weekly in duplicates as described by Sträuber et al. (2012).

TE concentrations were analyzed in duplicates at four sampling times (weeks 65, 77, 80, 84). Total element concentrations of TE and major elements were determined according to Schmidt et al. (2013). Daily concentrations of cobalt, manganese, molybdenum, nickel, and zinc between sampling times were estimated by a mass-conservative reactor model with daily feeding according to

$$\begin{array}{c} c_{t}=c_{t-1}+\frac{1}{\tau }\times (c_{\mathrm{Inflow}}-c_{t-1}) \end{array}$$

with concentration *c*_t_ of the respective TE at day *t*, concentration at the preceding day *c*_t-1_, total TE concentration in the inflow *c*_Inflow_ (summing over substrate, iron additive, and TE mixture), and retention factor τ given by the volume of the fluid reactor content divided by the daily exchanged fluid volume *V*_Reactor_/*V*_Exchange_. Goodness of model fit for one TE was evaluated as the mean deviation of model predictions *f_i_* from *n* measurements *y_i_*, relative to the measurement mean $\bar{y}:GF_{\mathrm{TE}}=\frac{1}{n}\sum_{i=1}^{n}|f_{i}-y_{i}|/\bar{y}$. A retention factor of 25 as calculated from the applied feeding regimen led to a reasonable fit with experimental data obtained before TE deprivation (mean *GF* over all TEs of 0.09), but not for those obtained after TE deprivation (mean *GF* of 0.14). This is likely due to the limitation of the analysis method for very low TE concentrations, which were reached at the end of the experiments. Hence, we used the modeled TE concentrations (Supplementary Data Sheet 2) for further statistical analysis.

### Extraction of Nucleic Acids and PCR

Samples for the extraction of nucleic acids were taken twice per week (three replicates of 2 mL reactor content) and stored at -80°C until DNA/RNA extraction. For DNA extraction, the PowerSoil DNA Isolation Kit (MoBio Laboratories Inc., USA) was used and DNA was finally eluted in 50 μL elution buffer. For RNA extraction, the ZR Soil/Fecal RNA Microprep Kit (Zymo Research, Germany) was used and the RNA was eluted in 40 μL elution buffer. The quality of the nucleic acids was checked by agarose gel electrophoresis. DNA was quantified with a NanoDrop^®^ ND-1000 UV-Vis spectrophotometer (ThermoFisher Scientific, Germany) and RNA was quantified after staining with RiboGreen (Invitrogen, USA) using a NanoDrop 3300 fluorimeter (ThermoFisher Scientific, Germany). Total RNA was converted to cDNA using the RevertAid^TM^ H Minus First Strand cDNA Synthesis Kits (Fermentas, Germany) and applying random hexamer primers. Aliquoted DNA and cDNA samples were kept at -20°C until further analysis. For PCR amplification of bacterial 16S rRNA gene fragments, the primers UniBac27F (5′-GAG TTT GAT CMT GGC TCA G-3′) and Univ1492R (5′-TAC GGY TAC CTT GTT ACG ACT T-3′) were used (according to Lane, 1991). The cycling program included an initial denaturation step of 4 min at 94°C, 30 cycles of 45 s at 94°C, 1 min at 58°C, 2 min at 72°C, and a final elongation step of 20 min at 72°C. For the amplification of *mcr*A gene fragments, the primer set (mlas/mcrA_rev) and the cycling program described by Steinberg and Regan (2008) were applied. PCR was carried out in 12.5-μL reaction mixtures. The reaction mixtures for both genes contained 1.0 μL (5 ng) genomic DNA or cDNA, respectively, 0.5 μL (2.5 pmol) of each primer (Eurofins Genomics, Ebersberg, Germany), 0.5 μl DMSO and 6.25 μL of *Taq* Master Mix (Qiagen, Hilden, Germany).

### T-RFLP Analysis of 16S rRNA and *mcrA* Amplicons

The T-RFLP analysis of bacterial and methanogenic communities using FAM-labeled PCR products was done as described previously (Sträuber et al., 2012; Lucas et al., 2015). PCR product quality was checked by agarose gel electrophoresis and amplicons were purified with SureClean (Bioline, Luckenwalde, Germany). Purified PCR products were quantified after electrophoresis in 1.5% agarose gels with ethidium bromide staining using the GeneTools program (Syngene, Cambridge, UK). The purified PCR products were digested with restriction endonucleases purchased from New England Biolabs (Schwalbach, Germany). The *mcr*A amplicons were digested with *Mwo*I and the 16S rRNA amplicons with *Rsa*I, using 2 units of the respective enzyme for digesting 10 ng of PCR product at 37°C overnight. The subsequent T-RFLP analysis was done for the *mcrA* amplicons with the GeneScan^TM^-500Rox^TM^ (Applied Biosystems, USA) as fragment size standard and for the 16S rRNA amplicons with the MapMarker1000 (BioVentures Inc., USA). Resulting electropherograms were analyzed by using the GeneMapper 5 software (Applied Biosystems) and processed according to Abdo et al. (2006). To differentiate between peaks and background, signals with low peak areas were removed according to eight times the standard deviation.

### Sequencing of *mcrA* and 16S rRNA Amplicons

Cloning, sequencing, and identification of the *mcrA* amplicons were conducted as described by Lucas et al. (2015). The obtained partial *mcrA* sequences were deposited in GenBank under accession numbers KU179685-KU179691.

The bacterial communities of both reactors at two sampling times (week 76, 80) were analyzed by amplicon sequencing of the bacterial 16S rRNA genes using the 454 pyrosequencing platform GS Junior (Roche) as described previously (Ziganshin et al., 2013). Raw sequence data were processed with MOTHUR (Schloss et al., 2009). The workflow was based on 454 SOP^1^. After extracting FASTA and quality files out of the SFF file, the trim.seqs command was run by defining barcodes and primers (maxambig = 0, maxhomop = 8, bdiffs = 1, pdiffs = 2, minlength = 150, qwindowaverage = 35, qwindowsize = 50), producing a trimmed FASTA file. After running unique.seqs and aligning the sequences (reference = silva.bacteria.fasta), these sequences were screened (start = 1044, optimize = end) and filtered (vertical = T, trump=.). Chimeras were deleted using the chimera.uchime (dereplicate = T) command based on the UCHIME algorithm (Edgar et al., 2011) and phylogenetic classification of the sequences was done based on the SILVA database (Quast et al., 2013) (cutoff = 50). Operational taxonomic units (OTU) were defined using the dist.seqs command with a cutoff of 0.03. Finally, the OTUs were classified, a list of representative sequences for each OTU was compiled, and rarefaction curves were calculated with the rarefaction.single command. De-multiplexed sequences from each sample were deposited under the EMBL-EBI accession number PRJEB11824^2^.

### Statistical Analyses

Multivariate statistical analysis of normalized T-RFLP peak tables was executed using the R package “vegan” (Oksanen et al., 2011). Clustering and non-metric multidimensional scaling (nMDS) analyses were performed based on the Bray-Curtis dissimilarity index (Bray and Curtis, 1957). The function “envfit” was used to identify the abiotic parameters and the terminal restriction fragments (T-RF) which shaped the community most. The significance was assessed by 1000 permutations.

## Results

### Decline of TE Concentrations and the Effect on Reactor Performance

**FIGURE 1:**
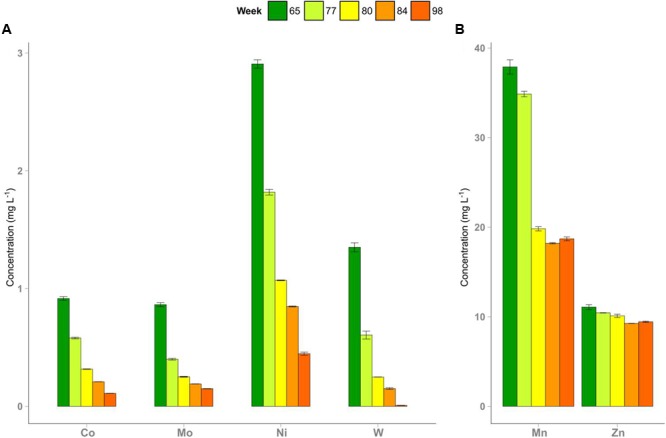
**Depletion of trace elements in reactor R2 after omitting the trace element (TE) supply and decreasing the amount of the iron additive in week 72.** Concentrations of cobalt, molybdenum, nickel, and tungsten **(A)**, manganese and zinc **(B)** are shown. Error bars indicate minimum and maximum values of duplicate measurements.

In week 72, TE deprivation in R2 was started and continued until week 93. After omitting the TE solution and reducing the amount of the iron additive in R2, total concentrations of most TE decreased (**Figure 1**) depending on whether their main sources were the TE solution, the iron additive or the substrate. Average TE concentrations (week 50 till week 93) in R1 were as follows (in mg L^-1^): cobalt 0.92 (±0.04), iron 987.00 (±62.96), manganese 38.75 (±6.62), molybdenum 0.81 (±0.04), nickel 3.15 (±0.14), tungsten 1.23 (±0.06), and zinc 10.53 (±0.68). These average concentrations resembled those in R2 during the period with full TE supply. These TE were supplied to the reactors as components of the substrate (mainly zinc), the iron additive (mainly iron and manganese), and the TE solution (mainly cobalt, molybdenum, nickel, and tungsten). Most TE (cobalt, molybdenum, nickel, tungsten) concentrations declined over time. Cobalt, molybdenum, nickel, and tungsten were present at concentrations of 1-3 mg L^-1^ before the start, which then dropped below 1 mg L^-1^ in R2 during the experiment. Manganese (40 mg L^-1^) was present at a higher concentration at the beginning and declined to 20 mg L^-1^. Zinc dropped from a concentration of 11 to 9 mg L^-1^. Concentrations of the latter two elements decreased until week 84 and remained stable thereafter. The TE concentrations in R1 showed no significant changes during the experiment.

**FIGURE 2:**
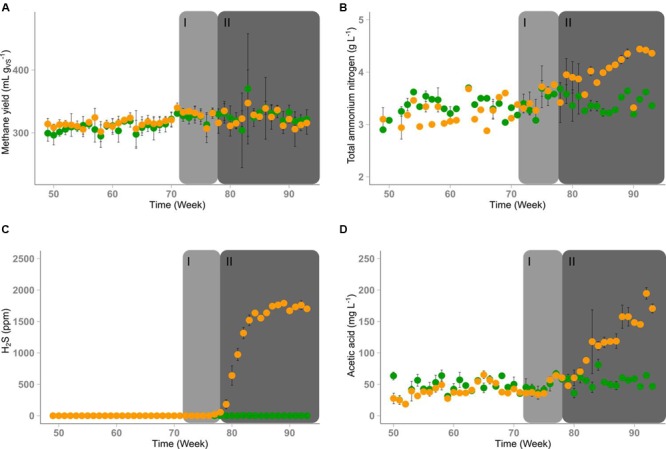
**Time course of process parameters of reactor R1 (green) and reactor R2 (orange).** Depletion of TEs started in time period I (week 72-78, marked in light gray). Significant changes of some process parameters were observed in time period II (after week 78, marked in dark gray). **(A)** Average methane yields, error bars indicate standard deviation (*n* = 5). **(B)** Concentration of TAN in the reactor content, error bars indicate minimum and maximum values when measured twice per week. **(C)** Average hydrogen sulfide concentrations in the biogas, error bars indicate standard deviation (*n* = 5). **(D)** Average acetic acid concentrations in the reactor content, error bars indicate standard deviation (*n* = 6).

The process parameters of both reactors are shown in **Figure 2** and Supplementary Figures S1-S15. Initially, R1 and R2 were operated in parallel with DDGS as substrate and adequately supplied with TE for 72 weeks. Due to the iron amendment, no hydrogen sulfide was detected in the gas produced in both reactors. During the period of identical operation, the average methane yields of R1 with 315 (±20) mL g_V S_^-1^ and R2 with 314 (±20) mL g_V S_^-1^ (standard deviation in parentheses) showed no significant shifts as well as the pH values in both reactors with 7.74 (±0.04) for R1 and 7.72 (±0.02) for R2. The biogas of both reactors contained 57% (±2%) methane and 43% (±2%) carbon dioxide, the average organic acids/alkalinity ratio was 0.14 (±0.01) in both reactors and the TAN concentrations were 3.4 (±0.2) mg L^-1^ and 3.3 (±0.2) mg L^-1^ in R1 and R2, respectively. Acetate concentrations were very low with 50 (±14) mg L^-1^ in R1 and 40 (±14) mg L^-1^ in R2. The sum concentrations of propionate and *n*-butyrate were mostly below 10 mg L^-1^. For R1 without TE deprivation, these process parameters did not change during the entire experimental period.

Four weeks after the TE solution was omitted in R2 and the supply of the iron additive was decreased, a brighter color of the digestate and a stronger sulfidic odor of the biogas were noticed. The formation of hydrogen sulfide reached a concentration of 1700 ppm in the gas phase of R2 after eight weeks of TE deprivation (week 80; **Figure 2C**). The hydrogen concentration in the gas phase increased from 70 to 110 ppm on average where it remained until the end of the experiment in R1 whereas it increased to more than 400 ppm in R2 after week 87 (Supplementary Figure S7). Although the biogas yield of R2 did not change (Supplementary Figure S2), the methane content of the biogas produced in R2 decreased over time (Supplementary Figure S5). However, the slight decrease of the methane content in R2 did not significantly affect the methane yield (**Figure 2A**). The unchanged pH value (Supplementary Figure S3) indicated that there was no strong process imbalance. In contrast, the total organic acid concentration increased from 1.3 to 1.6 g L^-1^ (Supplementary Figure S4). This change seemed to originate from the increase of the acetate concentration up to 200 mg L^-1^ (**Figure 2D**). Concentrations of propionic, butyric, valeric, and caproic acids did not differ significantly between both reactors (Supplementary Figures S8–S13). After TE deprivation, the final TAN concentration in R2 reached 4.5 g L^-1^ (**Figure 2B**). For the sake of clarity, the experimental time starting from week 72 was divided into a period without visible effects (I **–** until week 78) and a period with visible effects (II **–** from week 79 on).

### Effect of the TE Deprivation on the Microbial Community Composition

**FIGURE 3:**
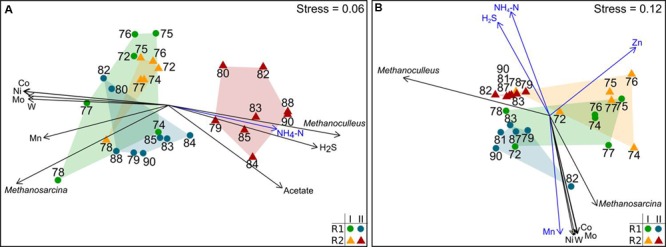
**Non-metric multidimensional scaling (nMDS) plots of T-RFLP profiles of *mcrA* transcripts (A)** and *mcrA* genes **(B)**. Data from reactor R1 (green dots: period I, week 72–78; blue dots: period II, after week 78) and reactor R2 (orange triangles: period I, week 72-78; red triangles: period II, after week 78) are shown. Data points are labeled by week of sampling. Plots are based on the Bray-Curtis dissimilarity index. Environmental factors and T-RF shaping the community profiles most are shown as vectors (blue: *p* < 0.01; black: *p* < 0.001).

The community composition and dynamics were determined by T-RFLP fingerprinting of *mcrA* and bacterial 16S rRNA amplicons. The resulting *mcrA* T-RF profiles indicated changes in the methanogenic community composition (DNA-based profiles) and microbial activity (cDNA-based profiles). **Figure 3** shows nMDS plots for *mcr*A on cDNA and DNA level along with process parameters significantly associated with community shifts. The underlying T-RFLP profiles are shown in the Supplementary Figures S18–S23. The active methanogenic community based on *mcrA* transcripts was similar in R1 and R2 during the first six weeks after starting the TE depletion (period I). After week 78, the composition of *mcrA* transcripts in R2 was remarkably different. The effect of TE deprivation was more distinct on the cDNA level compared to the DNA level. The methanogenic communities of R1 and R2 were clearly dominated by two sequence types seen as major T-RF assigned to *Methanosarcina* sp. (T-RF 122) and *Methanoculleus* sp. (T-RF 113). The relative abundances of these T-RF in R1 and R2 were similar until week 72 indicating a high stability of the methanogenic communities in both reactors. In period II, proportions of *mcr*A transcripts of *Methanoculleus* sp. rose up to 70% whereas *mcrA* transcripts of *Methanosarcina* sp. dropped to 17% in R2. The relative abundance of *mcr*A transcripts from *Methanosarcina* sp. was positively correlated with the concentrations of cobalt, manganese, molybdenum, nickel and tungsten, whereas *mcr*A transcription of *Methanoculleus* sp. rose in parallel with rising acetate, TAN and hydrogen sulfide concentrations (**Figure 3A**). Similar dependencies were found for the methanogenic community composition based on DNA data (**Figure 3B**).

**FIGURE 4:**
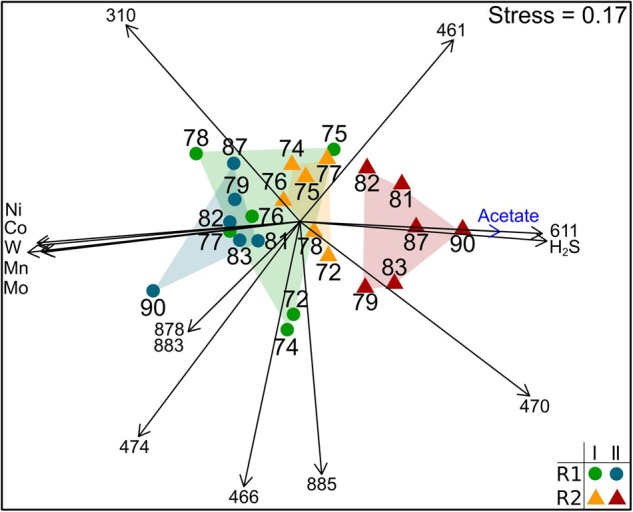
**Non-metric multidimensional scaling plot of T-RFLP profiles of bacterial 16S rRNA genes of reactor R1 (green dots: period I, week 72–78; blue dots: period II, after week 78) and reactor R2 (orange triangles: period I week 72-78; red triangles: period II after week 78).** Data points are labeled by week of sampling. Plots are based on the Bray-Curtis dissimilarity index. Environmental factors and T-RF shaping the community composition most are shown as vectors (blue: *p* < 0.01; black: *p* < 0.001).

The bacterial community composition based on 16S rRNA genes was stable in R1 during the whole experiment and in R2 until week 78 (period I – **Figure 4**). The community profiles showed some dominant T-RF including T-RF 166, 310, 461, 470, and 570 (Supplementary Figures S22 and S23). The bacterial community in R2 changed after week 78. Changes of various bacterial T-RF were found with reduced concentrations of the same TE that influenced the methanogenic community and with rising concentrations of acetate and hydrogen sulfide (**Figure 4**). For instance, the relative abundance of T-RF 310 dropped from 30 to 8% and T-RF 474 disappeared in R2 during TE deprivation. In contrast, the proportion of T-RF 461 was stable in R2 whereas it dropped in R1.

**FIGURE 5:**
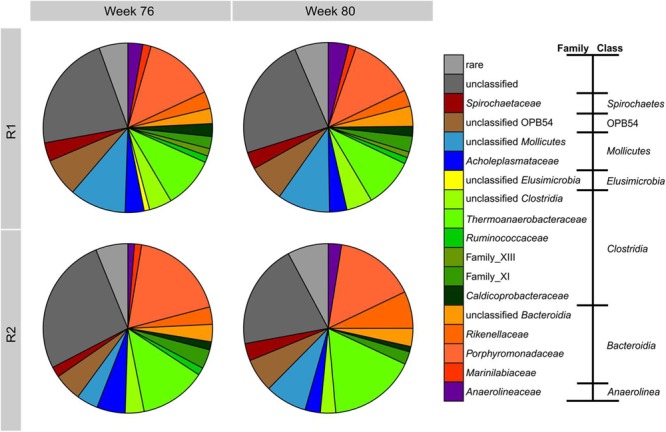
**Phylogenetic composition based on 454 amplicon sequencing of bacterial 16S rRNA genes from samples of R1 and R2 in weeks 76 and 80.** Bacterial community composition is shown at the family level sorted according to the class level. Families with maximum abundances below 1% are summarized as rare.

The bacterial community composition was determined by amplicon sequencing resulting in 55165 high quality reads, which were assigned to 1661 OTUs (97% similarity cutoff). Approximately 14000 reads were obtained per sample. All samples had 317 OTUs in common and each sample included on average 150 unique OTUs. These commonalities and differences are illustrated in a Venn diagram (Supplementary Figure S17). The respective rarefaction curves (Supplementary Figure S16) and the OTU list with representative sequences and their phylogenetic affiliations are given in the Supplementary Material (Data Sheet 3). The phylogenetic composition of the bacterial communities on the family level is shown in **Figure 5**. The bacterial community composition of both reactors was stable between weeks 76 and 80 sharing the same major bacterial families. Coverage and diversity indices (Inverse Simpson Index, Shannon Index, Shannon Evenness Index) are given in Supplementary Table S1 showing a comparable diversity and evenness between the samples. The predominant class was *Clostridia* with *Thermoanaerobacteraceae* as the main family comprising at least 10% of the total bacterial community. All OTUs assigned to this family belonged to the genus *Gelria*. *Bacteroidia* represented by the families *Porphyromonadaceae* (88% of which were assigned to the genus *Proteiniphilum*) and *Rikenellaceae*, were the second dominant class followed by representatives of the class *Mollicutes*. During TE deprivation, the proportion of *Thermoanaerobacteraceae* in R2 increased up to 16% of the bacterial community. *Rikenellaceae* increased their relative abundance from 3 to 10% between week 76 and 80. The proportions of family_XIII (*Clostridia*), *Marinilabiaceae* (*Bacteroidia*), and *Ruminococcaceae* (*Clostridia*) decreased in R2 relative to R1.

## Discussion

Our study has shown that TE deprivation has a remarkable effect on the methanogenic community in anaerobic digesters treating DDGS. The decline of nickel, cobalt, molybdenum, tungsten, and manganese most strongly influenced the activity ratio of the dominant methanogens *Methanosarcina* and *Methanoculleus*. This observation is based on the total TE concentrations, which do not provide information on the bioavailable fraction of the respective TE. The bioavailability of trace metals depends on their chemical speciation which is influenced by the reactor configuration and operating conditions such as pH value, redox potential, temperature and hydraulic retention time (Thanh et al., 2016). Soluble TE supplied with the feedstock can be converted to less bioavailable forms by adsorption, chelation/complexation or precipitation. Among the chemical processes affecting TE bioavailability, the precipitation of metal sulfides is the most critical one (Thanh et al., 2016). Thus, sulfide formed during the AD process can affect process performance not only due to the toxicity of hydrogen sulfide but also by impairing the bioavailability of essential trace metals. DDGS is a protein-rich substrate from which a high amount of sulfide is generated during AD (Gustavsson et al., 2011). Gustavsson et al. (2013) showed that only 10–20% of cobalt, an element that is in general easily accessible for microorganisms in AD, remained in a dissolved form when elevated sulfide concentrations were present. Nickel was entirely associated with organic matter or present as sulfide and had to be added regularly to remain bioavailable (Gustavsson et al., 2013).

In our experiment, most of the factors which determine trace metal speciation did not change except of TE supply and iron amendment. When we reduced the iron amendment in R2, the iron concentration and that of other TE contained in the iron additive decreased, while simultaneously the free sulfide concentration increased, indicated by a brighter color of the reactor sludge (i.e., less iron sulfide), a strong sulfidic odor and increasing H_2_S concentrations in the biogas. Consequently, we assume that the intensified precipitation of TE caused by the low solubility of metal sulfides reduced the TE bioavailability (Rickard and Luther, 2006), although we did not apply analytical methods which can determine different TE speciations such as sequential extraction (Thanh et al., 2016). Our results underline the importance of counter-measures against sulfide release and compensation of TE loss due to sulfide precipitation when protein-rich feedstock is treated in AD.

Besides sulfide, ammonia is readily generated during anaerobic degradation of protein-rich substrates such as DDGS. During TE deprivation, the TAN concentration in R2 increased, indicating stronger degradation of proteins. However, it is unlikely that the DDGS degradation was intensified under these conditions. It may be that due to the increasingly constrained TE situation, a considerable amount of microorganisms died and the emerging microbial biomass was degraded by the surviving bacteria. This assumption is supported by unpublished results in our lab showing increasing TAN concentrations under changing feeding conditions and a return to initial TAN concentrations after process adaptation. High ammonia concentrations are known to inhibit acetoclastic methanogens which are outcompeted by syntrophic acetate oxidizing bacteria (SAOB) under such conditions (Schnürer and Nordberg, 2008; Westerholm et al., 2015). Within the bacterial community, VFA degrading syntrophic bacteria are very important for the process equilibrium. High concentrations of VFA like butyrate, propionate, or acetate are detrimental for the biogas process and indicators of process imbalances, emphasizing the crucial role of syntrophic bacteria (Ahring et al., 1995; Nielsen et al., 2007). During our experiment, the acetate concentration in R2 increased from 50 to 170 mg L^-1^, whereas butyrate and propionate did not accumulate. This indicates that syntrophic propionate or butyrate degraders remained unaffected by declining TE concentrations. In contrast, acetoclastic methanogenesis as a direct acetate sink seemed to be inhibited. Acetate degradation by SAOB as a possible alternative did not compensate for this, resulting in increasing acetate concentrations. SAOB have low growth rates (Hattori, 2008) and therefore adapt only slowly to altered conditions. At the end of the experiment, the acetate concentration was still rising and we can only speculate if the community adaptation was still in progress.

The dominance shift from *Methanosarcina* to *Methanoculleus* was the main response of the methanogenic community to TE deprivation in our study. This shift occurred between 4 and 6 weeks after the start of the TE omission leading to a shortage of cobalt, manganese, nickel, tungsten, and zinc. Whereas *Methanosarcina* is a direct degrader of acetate and methylamine (Liu and Whitman, 2008), *Methanoculleus* utilizes CO_2_ and H_2_ or formate and acts as a syntrophic partner for VFA degraders and SAOB. Without TE deprivation, the relative proportion of *mcrA* transcripts indicated that both methanogens were similarly active. After starting the TE deprivation, a shift of the methanogenic community was observed on the activity level (*mcrA* transcripts), which was accompanied by only a minor shift in community composition (*mcrA* genes). Six weeks after starting the TE deprivation, the transcription rates of *mcr*A changed. *Methanoculleus* contributed a higher proportion of the overall *mcr*A transcripts than *Methanosarcina* sp. However, the overall methane yield did not change, suggesting that *Methanoculleus* increased its *mcrA* expression while *mcrA* expression in *Methanosarcina* decreased simultaneously.

All methanogenesis pathways rely on the methyl-CoM reductase, which depends on the nickel-containing cofactor F430 (Dey et al., 2010). Likewise, many hydrogenases possess a nickel-iron center (Thauer et al., 2010). Acetoclastic and methylotrophic pathways contain corrinoid iron-sulfur proteins (Burke and Krzycki, 1997; Ferguson et al., 2000; Svetlitchnaia et al., 2006) and accordingly require cobalt. Several other enzymes involved in acetoclastic methanogenesis depend on specific TE. Acetate kinase requires magnesium, which, however, can be replaced by manganese, cobalt or calcium (Aceti and Ferry, 1988; Miles et al., 2001), and the acetyl-CoA decarbonylase/synthase complex (ACDS) contains cobalt and nickel (Jablonski et al., 1993). Hydrogenotrophic methanogenesis is independent of corrinoid iron-sulfur proteins, suggesting a lower demand of cobalt. On the other hand, hydrogenotrophic methanogenesis has other specific TE demands. The formylmethanofuran dehydrogenase uses molybdenum or tungsten as central atom (Bertram et al., 1994; Vorholt et al., 1996) and tetrahydromethanopterin-*S*-methyltransferase contains cobalt (Lienard et al., 1996). These diverse demands explain the TE dependency of methanogens.

We hypothesize that *Methanosarcina* and *Methanoculleus* adapt differently to TE deprivation in order to cover their energy demands. Deprivation of cobalt should lead to shortage of corrinoid iron-sulfur proteins required for methane production from acetate and methyl compounds. Furthermore, these pathways need approximately three times more nickel than hydrogenotrophic methanogenesis, based on the number of nickel-dependent enzymes involved in the respective methanogenic pathways. Therefore, we assume that *Methanosarcina* switched to hydrogenotrophic methanogenesis to save cobalt and nickel and thus became a competitor of *Methanoculleus* for formate and hydrogen. However, the prevailing hydrogen concentrations in AD usually match the requirements of *Methanoculleus* more than those of *Methanosarcina*. *Methanosarcina* requires higher H_2_ concentrations than *Methanoculleus* (Thauer et al., 2008), which is able to consume H_2_ at partial pressure of as low as <10 Pa (Garcia et al., 2000) occurring in most AD processes. The weaker performance of *Methanosarcina* after TE deprivation can also be explained by the different energy conservation mechanisms occurring in methanogenesis by electron transport phosphorylation and flavin-based electron bifurcation (Thauer et al., 2008). Nickel plays an essential role as central atom of the hydrogenases involved. Hydrogenotrophic methanogens are able to replace their [NiFe] hydrogenases by nickel-free [Fe] hydrogenases (HMD), which are unique for methanogens (Thauer et al., 2010). It has been shown that a nickel-responsive transcriptional regulator upregulates HMD under nickel-limiting conditions (Afting et al., 2000). Thus, the activity of *Methanosarcina* depends on nickel bioavailability more strongly as there is no alternative to [NiFe] hydrogenases. Although *Methanosarcina* should have been particularly affected by TE deprivation due to its higher cobalt and nickel demands and as its *mcrA* expression was indeed downregulated compared to *Methanoculleus*, its abundance decreased only slightly. In contrast, *Methanoculleus* sp. showed relatively higher *mcrA* expression, which can be taken as a sign of higher activity. However, *Methanoculleus* did not outcompete *Methanosarcina*.

We speculate that the ability of *Methanosarcina* to switch between methanogenic pathways enabled it to enter the niche of the strictly hydrogenotrophic methanogen *Methanoculleus*. We assume that the activity of SAOB increases at higher acetate concentrations as they prevail after inhibition of the acetoclastic methanogenesis. Additionally to acidogenic and acetogenic bacteria, SAOB provide formate and H_2_, which requires an increased metabolic activity of hydrogenotrophic methanogens such as *Methanoculleus*. As we have no information on hydrogen or formate concentrations in the liquid phase it was not possible to prove this hypothesis. One can speculate that *Methanoculleus* has to invest more energy to cope with TE limitation, for instance by increasing the production of TE transporters. This might explain why *Methanoculleus* did not overgrow *Methanosarcina* although it showed higher methanogenic activity.

## Conclusion

Slowly increasing TE deficits did not change the reactor efficiency as indicated by stable biogas and methane yields. Nevertheless, increasing TAN and acetate concentrations pointed at microbial community shifts which might affect reactor performance on the long run. Shifts within the methanogenic community were less visible in composition (*mcrA* genes) than in activity (*mcrA* transcripts), particularly with the two most abundant genera *Methanosarcina* and *Methanoculleus*. The bacterial composition changed only slightly suggesting a higher stress tolerance of the bacterial community due to a higher metabolic versatility. Our results confirm the importance of sufficient TE supply to keep the activity of the “heavy duty” methanogen *Methanosarcina* (De Vrieze et al., 2012). In contrast, *Methanoculleus* can cope better with limiting TE concentrations and keep the AD process stable under sub-optimal TE supply. However, whether the process efficiency can be kept in the long term cannot be predicted from this experiment.

## Author Contributions

BW, HS, JL, and SK designed the study and the experiments; BW and KG performed the experiments; BW, KG, HS and SK analyzed the data; FC designed and modeled the interpolation of trace element concentrations; BW, HS, JL, HH, and SK interpreted the data. BW and SK drafted the manuscript and HS, KG, FC, JL, and HH critically revised it. All authors approved the final version of the manuscript.

## Conflict of Interest Statement

The authors declare that the research was conducted in the absence of any commercial or financial relationships that could be construed as a potential conflict of interest.

## Footnotes

1 http://www.mothur.org/wiki/454_SOP

2 http://www.ebi.ac.uk/ena/data/view/PRJEB11824
