# Supplementary material for: Trace Elements Induce Predominance among Methanogenic Activity in Anaerobic Digestion
Source: Front Microbiol. 2016 Dec 16;7:2034. doi: 10.3389/fmicb.2016.02034 (PMC5160323; doi:10.3389/fmicb.2016.02034)
Supplement: Supplementary file 1 [file Data_Sheet_1.PDF]

Supplementary Material

Trace elements induce predominance among methanogenic activity in anaerobic digestion

Babett Wintsche, Karin Glaser, Heike Sträuber, Florian Centler, Jan Liebetrau, Hauke Harms, Sabine Kleinsteuber*

*** Correspondence:** sabine.kleinsteuber@ufz.de

1 Supplementary Data

The Excel file '**TE and Modelling**.**xlsx' (Data Sheet 2)** contains all data sets regarding trace elements (TE) in this study. In the spreadsheet 'Measured Cons', the measured concentrations determined at five sampling times (described in the Method section) of TE (Co, Fe, Mn, Mo, Ni, W, Zn) are given. The results for reactor R1 are visualized in bar charts while the results for reactor R2 are shown in the Results section. The TE contents of the fed DDGS, iron additive, water and TE mixture are shown in the spreadsheet 'Substrates' and the total fed TE concentrations are calculated in 'Fed TE'. In the following spreadsheets, the TE concentrations between the five sampling times were modeled for Co, Mn, Mo, Ni, W and Zn based on the equation given in the Methods section.

The Excel file '**OTU+Tax+Seq.xlsx' (Data Sheet 3)** shows the results of 454 amplicon sequencing of bacterial 16S rRNA genes at three sampling times for each reactor. In the spreadsheet 'OTUs', all operational taxonomic units (OTUs) and the number of reads assigned to the respective OTUs are shown. Their taxonomic affiliation is shown in the spreadsheet 'Taxonomy'. Column 'Size' describes the number of reads contained in the OTU. For each OTU, a representative sequence is given in the spreadsheet 'Representative sequences'.

2 Supplementary Figures and Tables

The **Supplementary Figures S1 – S15** show the complete dataset of the abiotic parameters from reactors R1 and R2 obtained in this study. Selected parameters are shown in the Results section. All parameters were determined as described in the Methods section.

Rarefaction curves and a Venn diagram resulting from amplicon sequencing data are shown in the **Supplementary Figures S16** and **S17**.

The relative T-RF abundances are given for the *mcr*A gene (cDNA 'mcrA cDNA MwoI' and DNA 'mcrA DNA MwoI') and the bacterial 16S rRNA gene (DNA '16S rRNA DNA RsaI'). T-RFLP analysis is described in the Methods section. The relative abundances for each reactor are visualized as bar charts in the **Supplementary Figures S18 – S23**.

2.1 Supplementary Figures

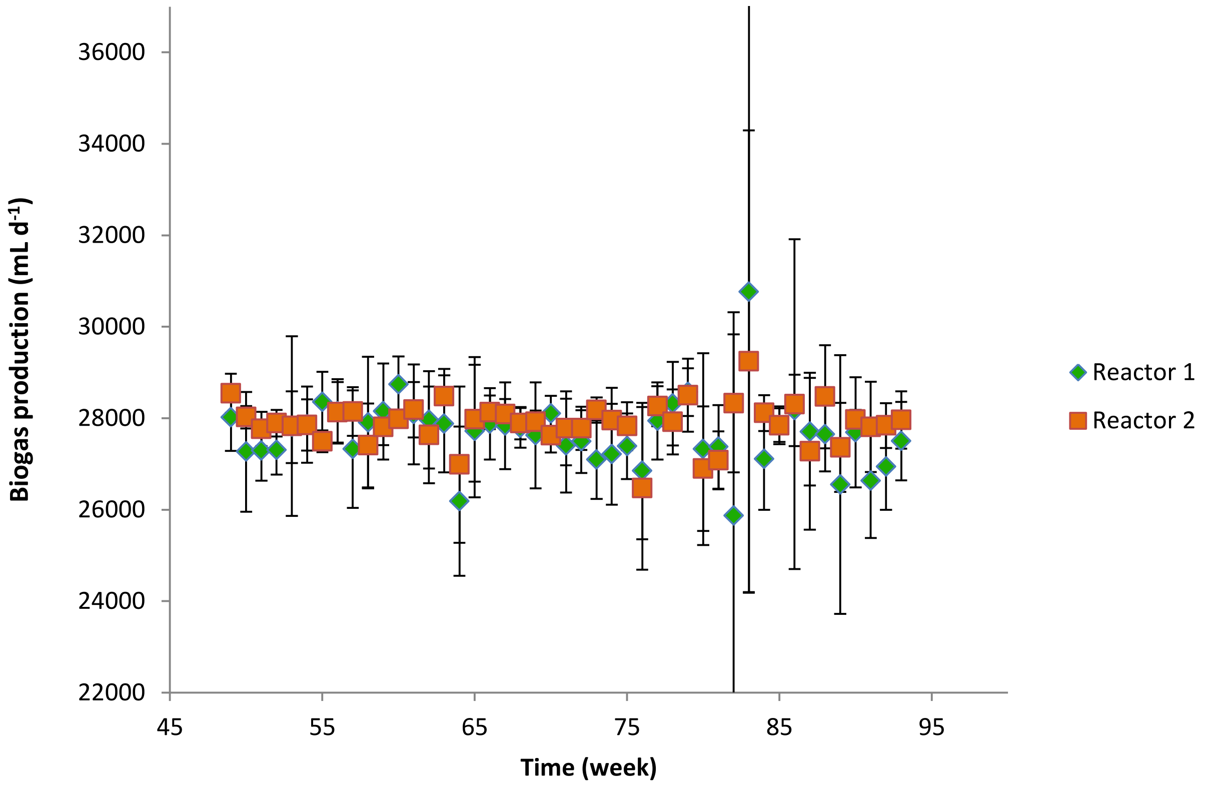

Figure S1. Average daily biogas production, error bars indicate standard deviation (n=5).

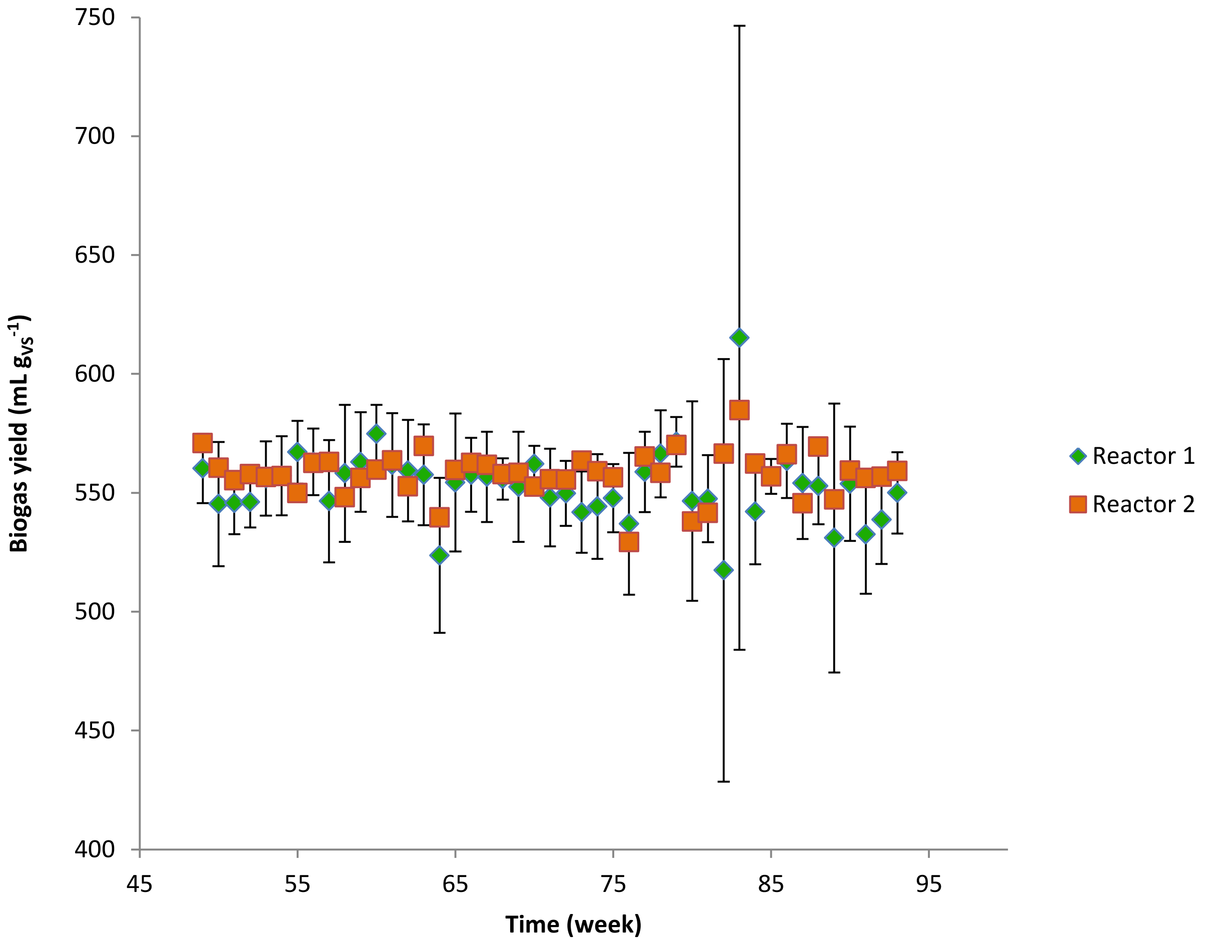

Figure S2. Average biogas yield, error bars indicate standard deviation (n=5).

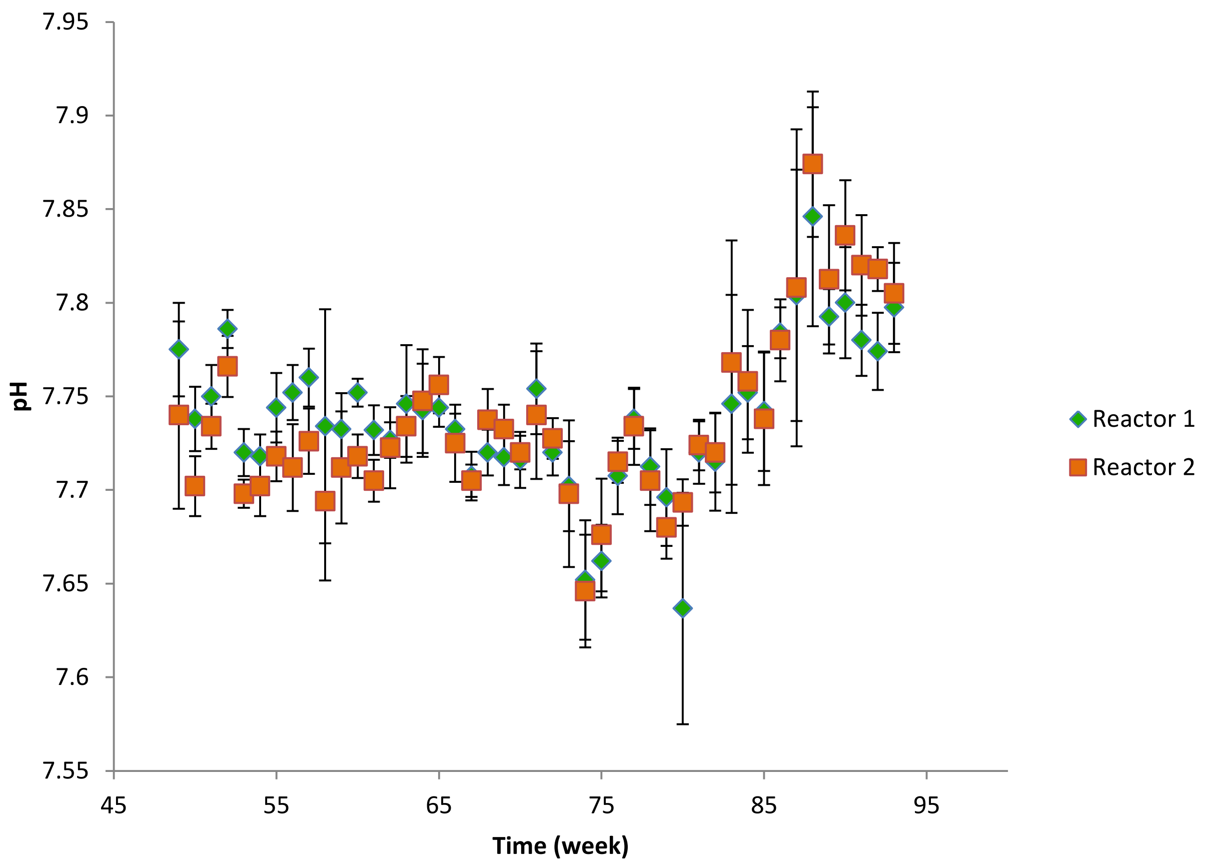

Figure S3. Average pH values of the reactor content, error bars indicate standard deviation (n=5).

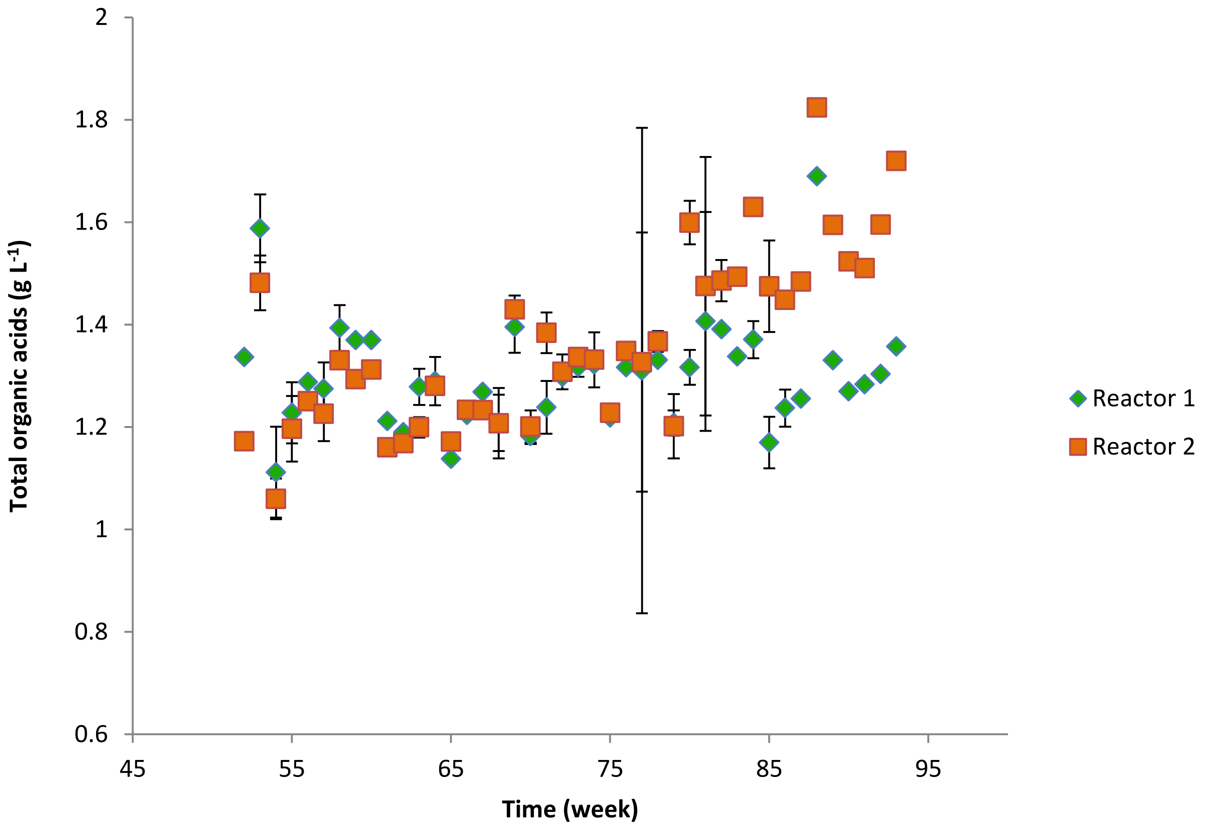

Figure S4. Average total organic acid concentration in the reactor content, error bars indicate standard deviation (n=6).

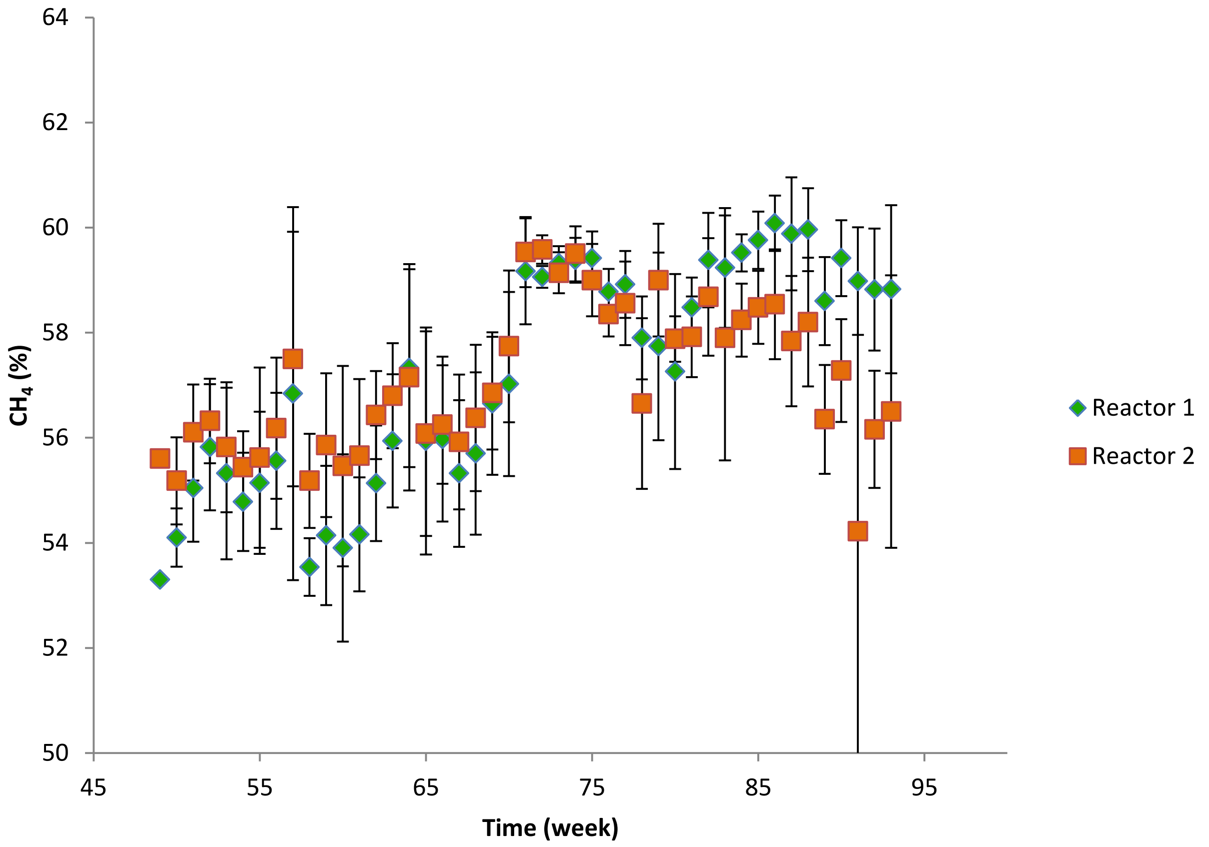

Figure S5. Average methane content of the biogas, error bars indicate standard deviation (n=5).

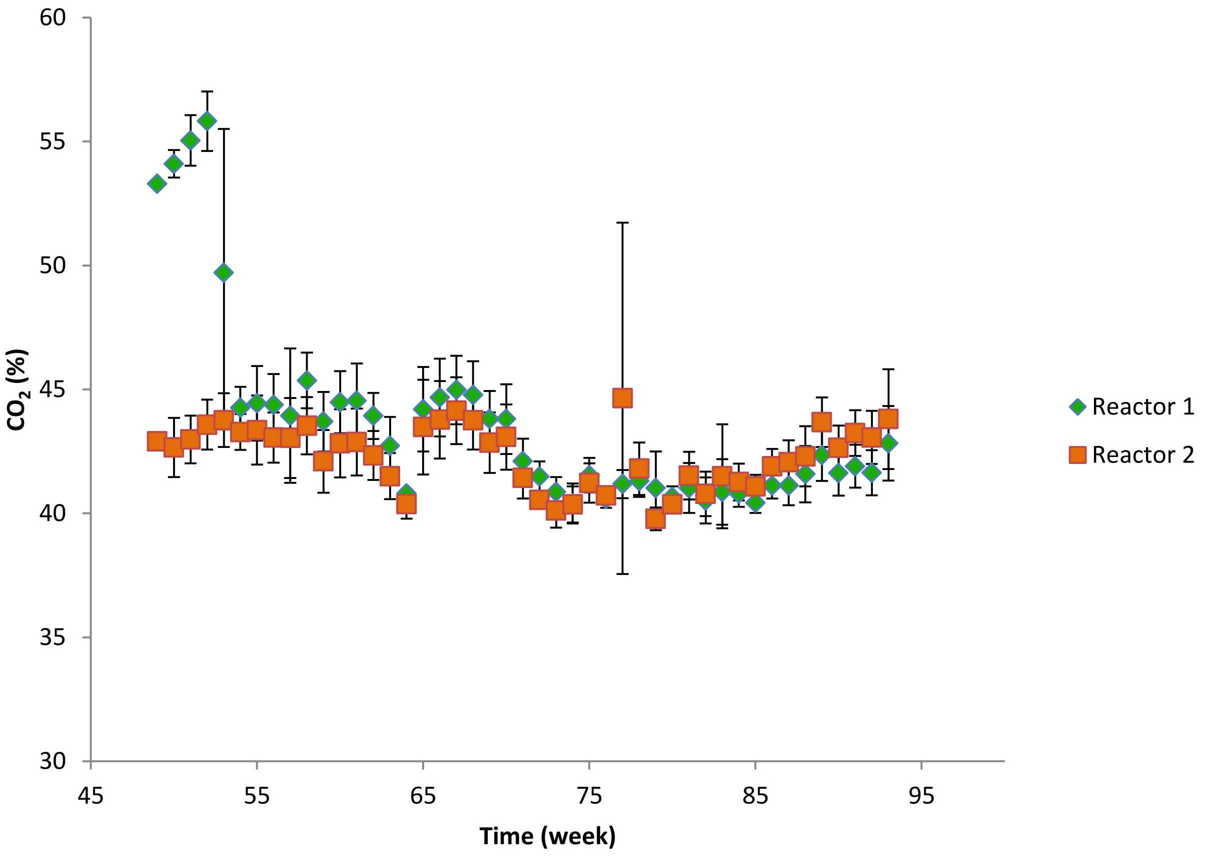

Figure S6. Average carbon dioxide content of the biogas, error bars indicate standard deviation (n=5).

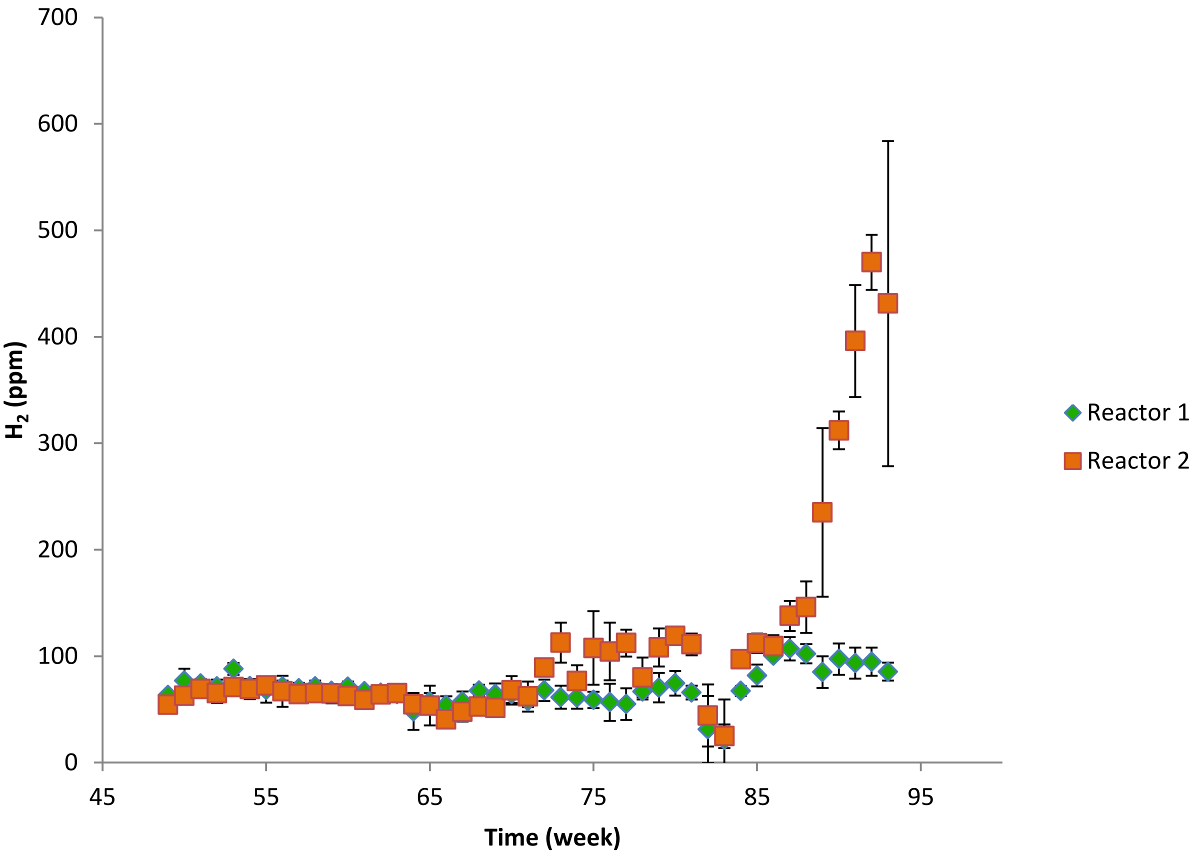

Figure S7. Average hydrogen content of the biogas, error bars indicate standard deviation (n=5).

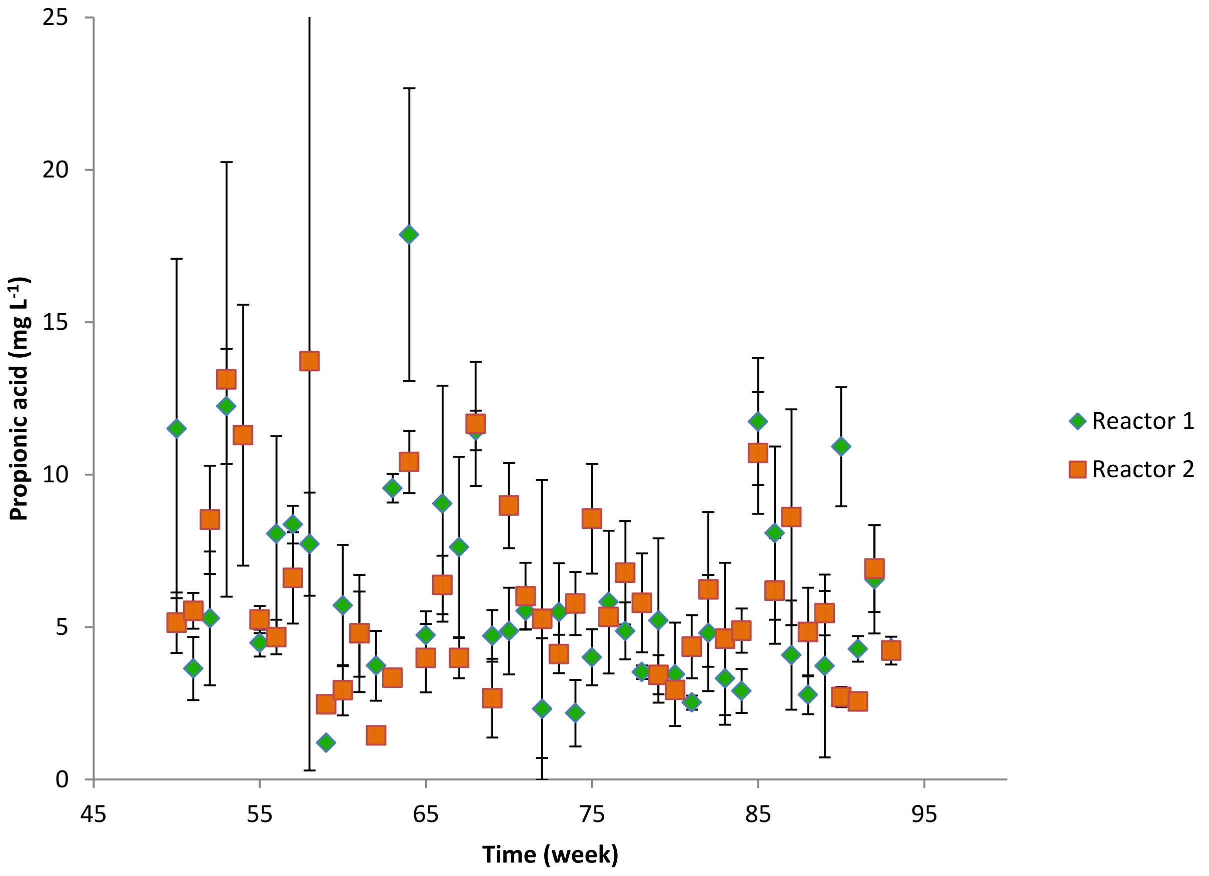

Figure S8. Average propionic acid concentration in the reactor content, error bars indicate standard deviation (n=6).

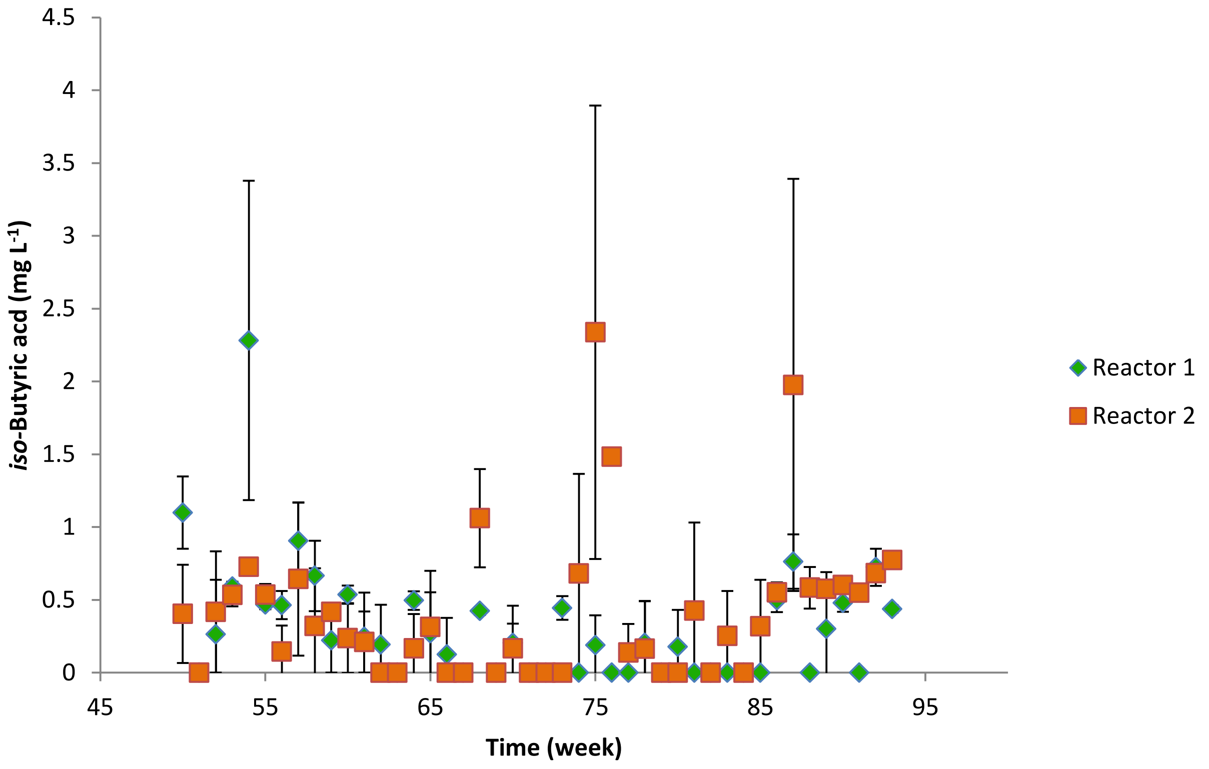

Figure S9. Average *iso*-butyric acid concentration in the reactor content, error bars indicate standard deviation (n=6).

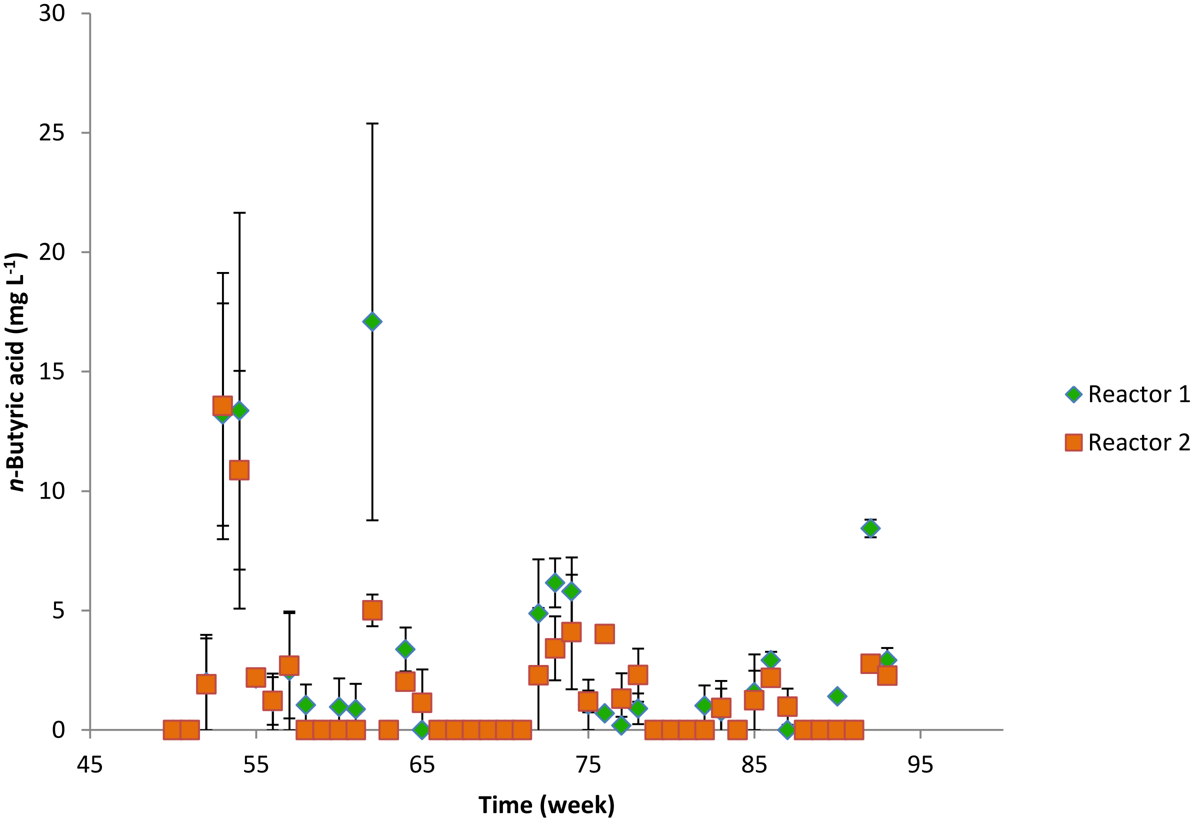

Figure S10. Average *n*-butyric acid concentration in the reactor content, error bars indicate standard deviation (n=6).

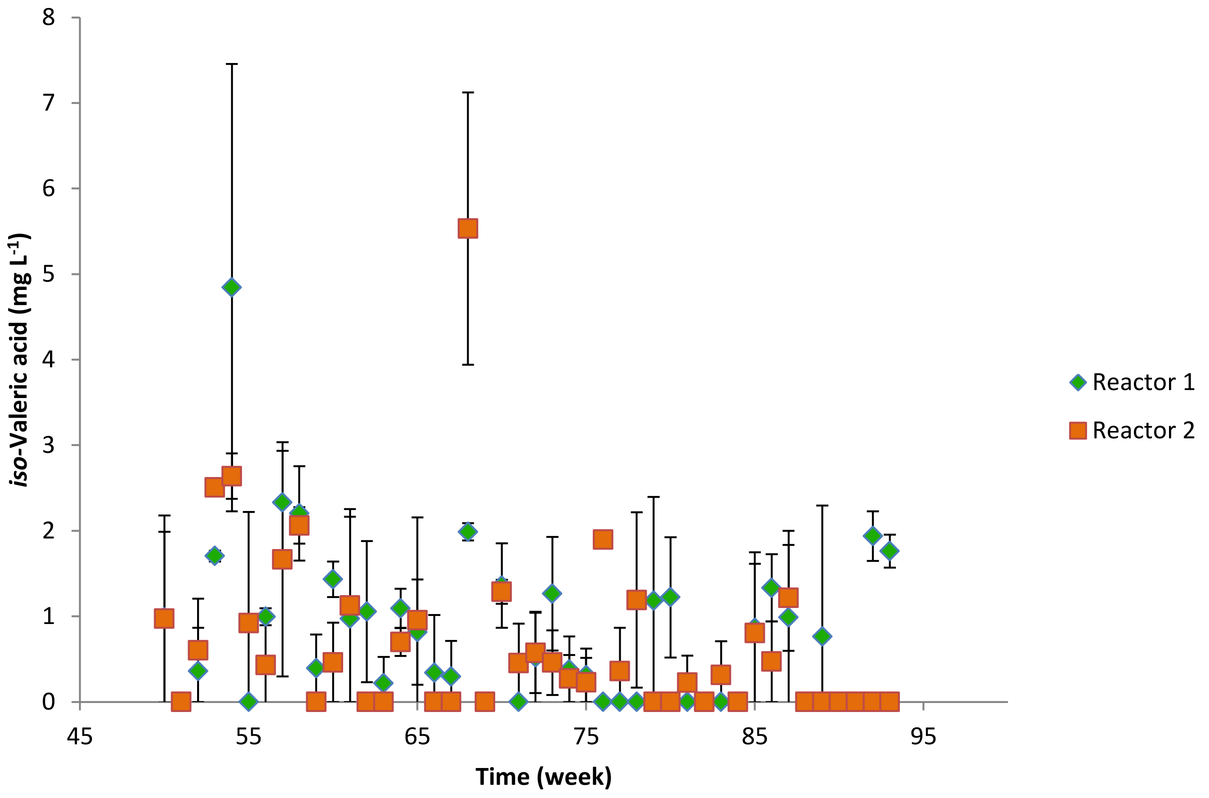

Figure S11. Average *iso*-valeric acid concentration in the reactor content, error bars indicate standard deviation (n=6).

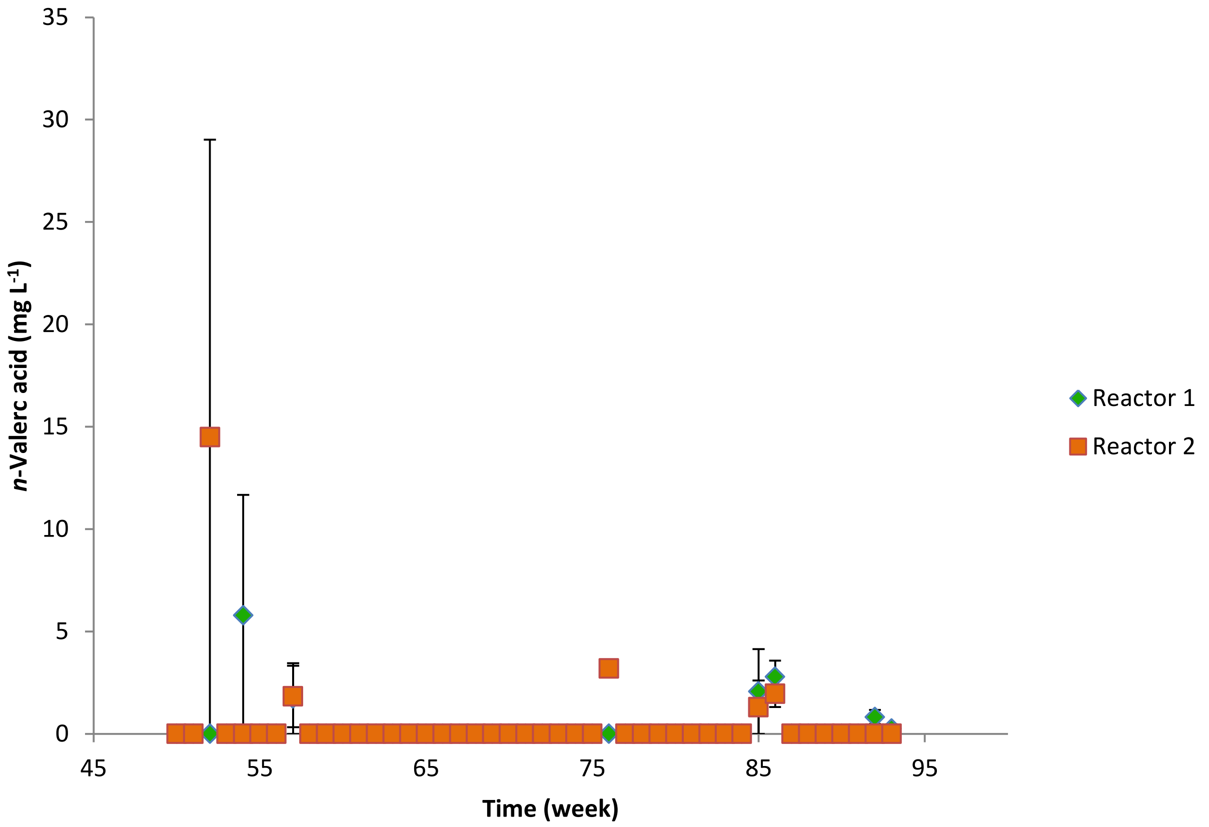

Figure S12. Average *n*-valeric acid concentration in the reactor content, error bars indicate standard deviation (n=6).

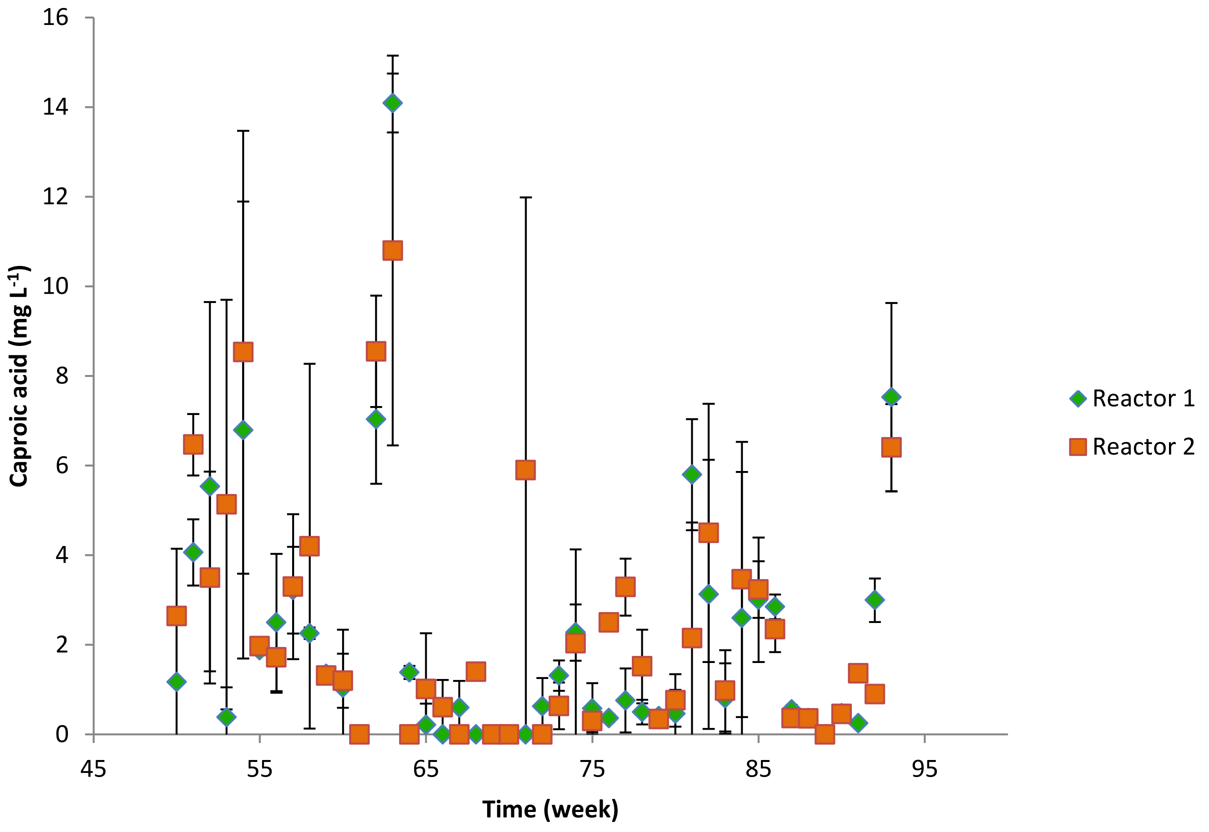

Figure S13. Average caproic acid concentration in the reactor content, error bars indicate standard deviation (n=6).

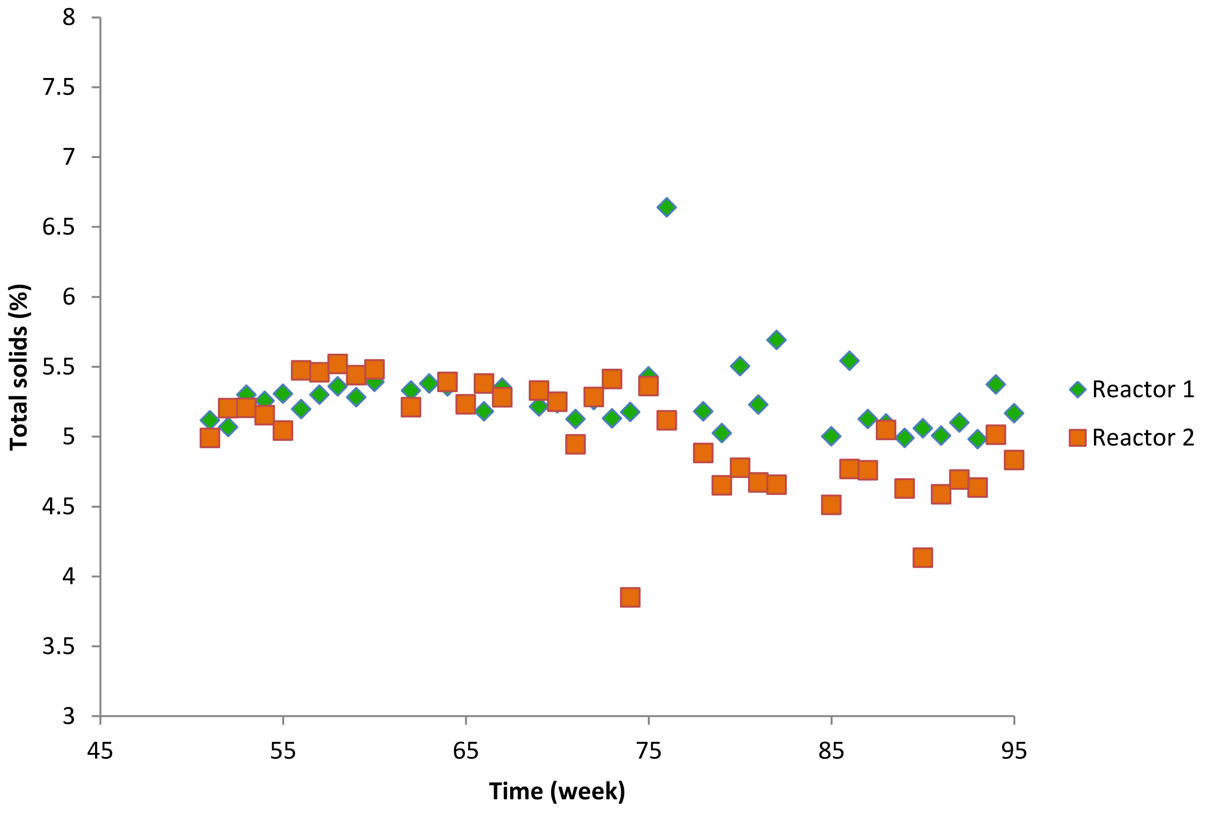

Figure S14. Total solids content of the digestate.

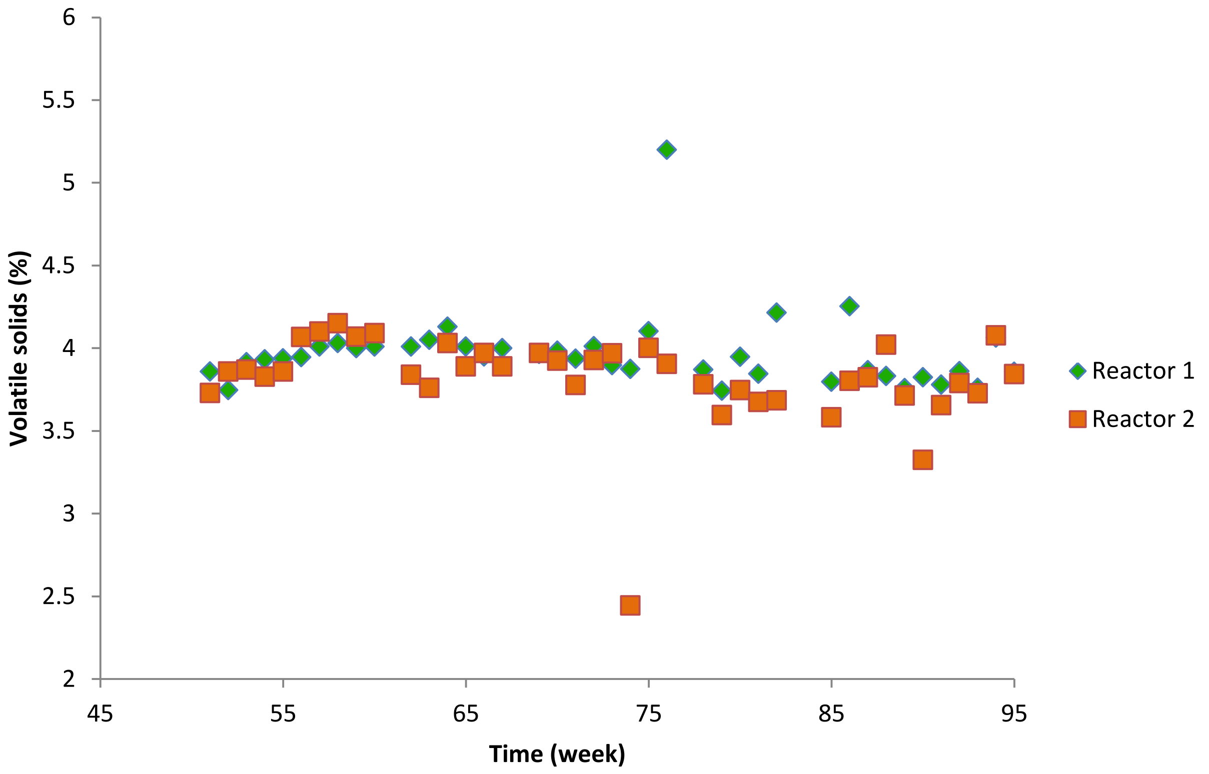

Figure S15. Volatile solids content of the digestate.

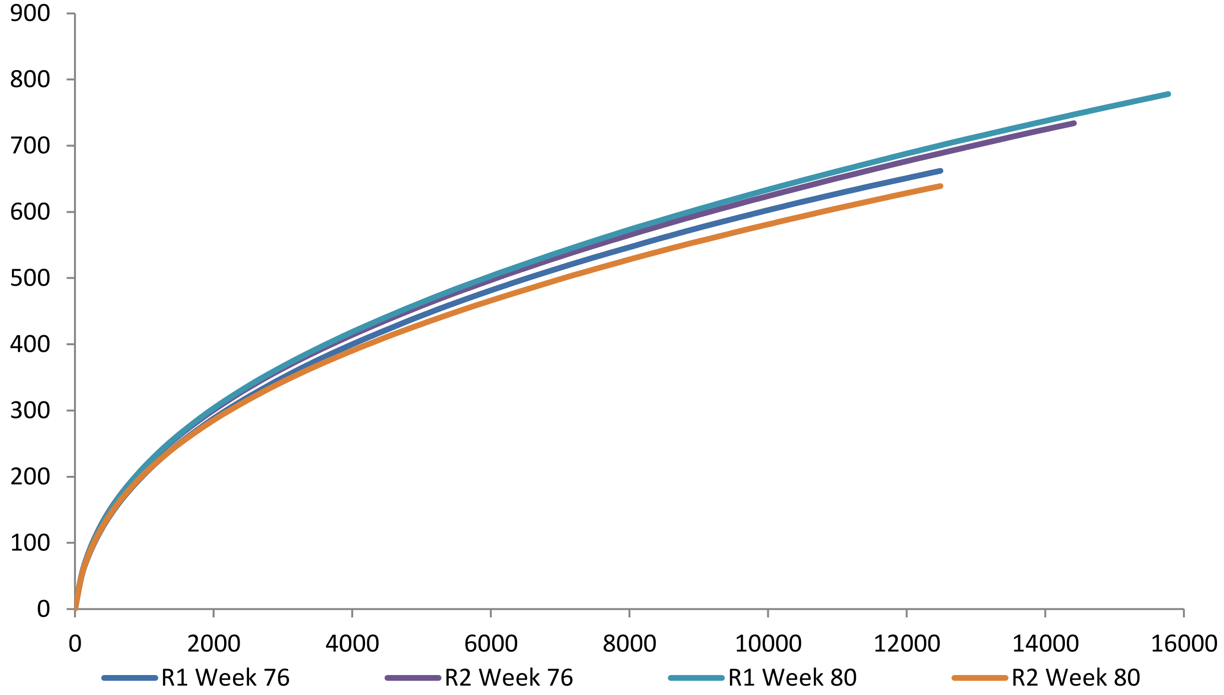

Figure S16. Rarefaction curves showing the coverage of the total bacterial richness based on 454 reads of bacterial 16S rRNA amplicons.

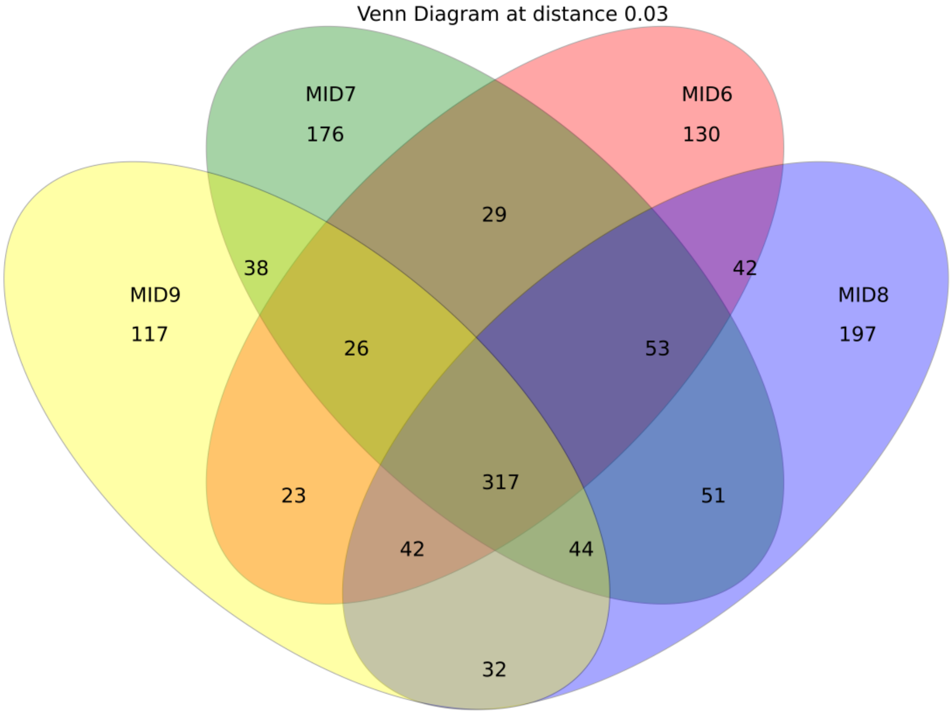

The number of species in group MID6 is 662
The number of species in group MID7 is 734
The number of species in group MID8 is 778
The number of species in group MID9 is 639
The number of species shared between groups MID6 and MID7 is 425
The number of species shared between groups MID6 and MID8 is 454
The number of species shared between groups MID6 and MID9 is 408
The number of species shared between groups MID7 and MID8 is 465
The number of species shared between groups MID7 and MID9 is 425
The number of species shared between groups MID8 and MID9 is 435
The number of species shared between groups MID6, MID7 and MID8 is 370
The number of species shared between groups MID6, MID7 and MID9 is 343
The number of species shared between groups MID6, MID8 and MID9 is 359
The number of species shared between groups MID7, MID8 and MID9 is 361
The total richness of all the groups is 1317

Figure S17. Venn diagram showing shared and unique OTUs between samples based on 454 reads of bacterial 16S rRNA amplicons. MID6 – Reactor R1, week 76; MID7 – Reactor R2, week 76; MID8 – Reactor R1, week 80; MID9 – Reactor R2, week 80.

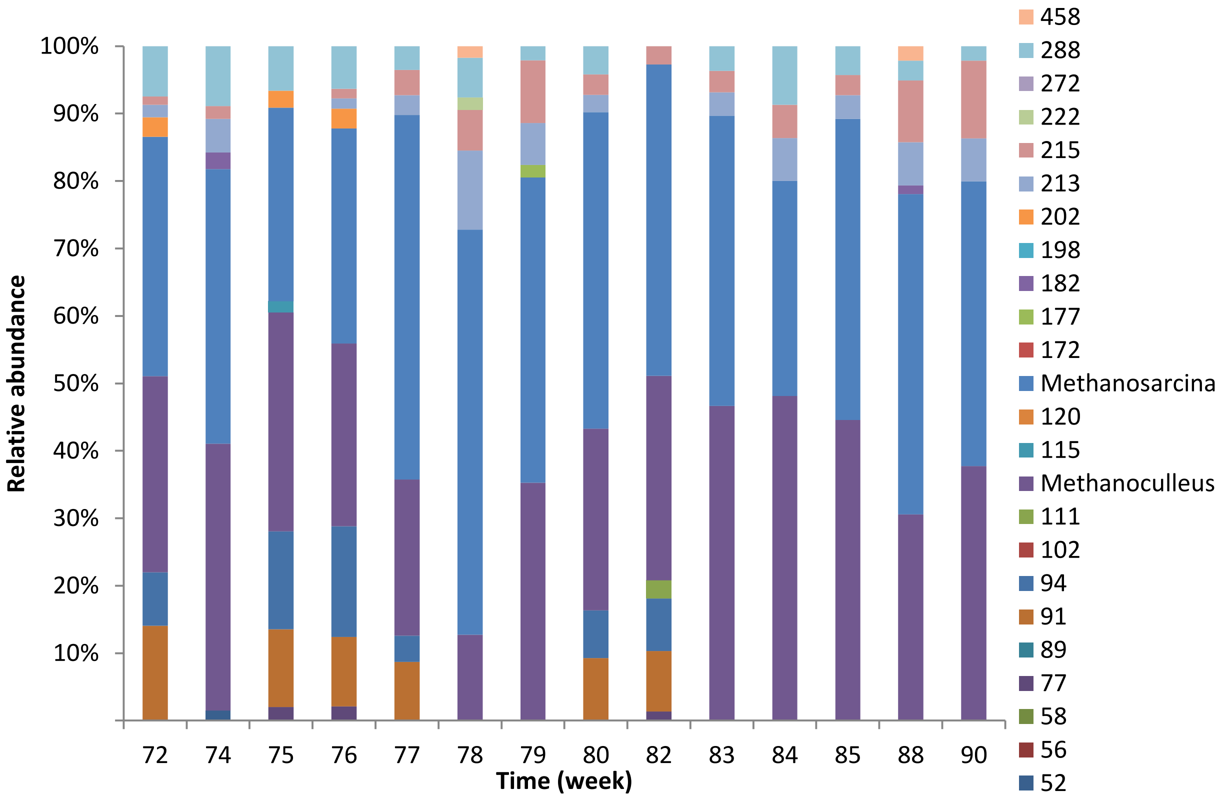

Figure S18. T-RFLP profiles of *mcr*A transcripts in samples from reactor R1.

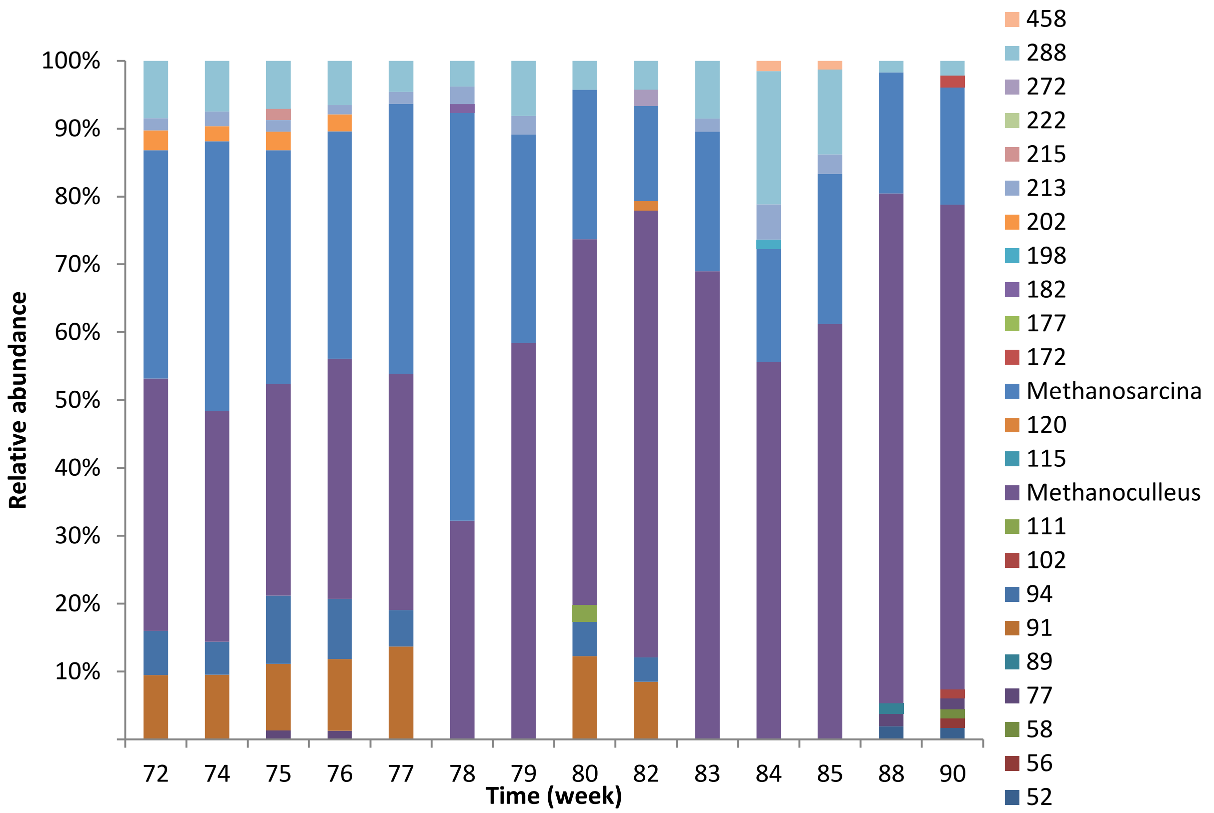

Figure S19. T-RFLP profiles of *mcr*A transcripts in samples from reactor R2.

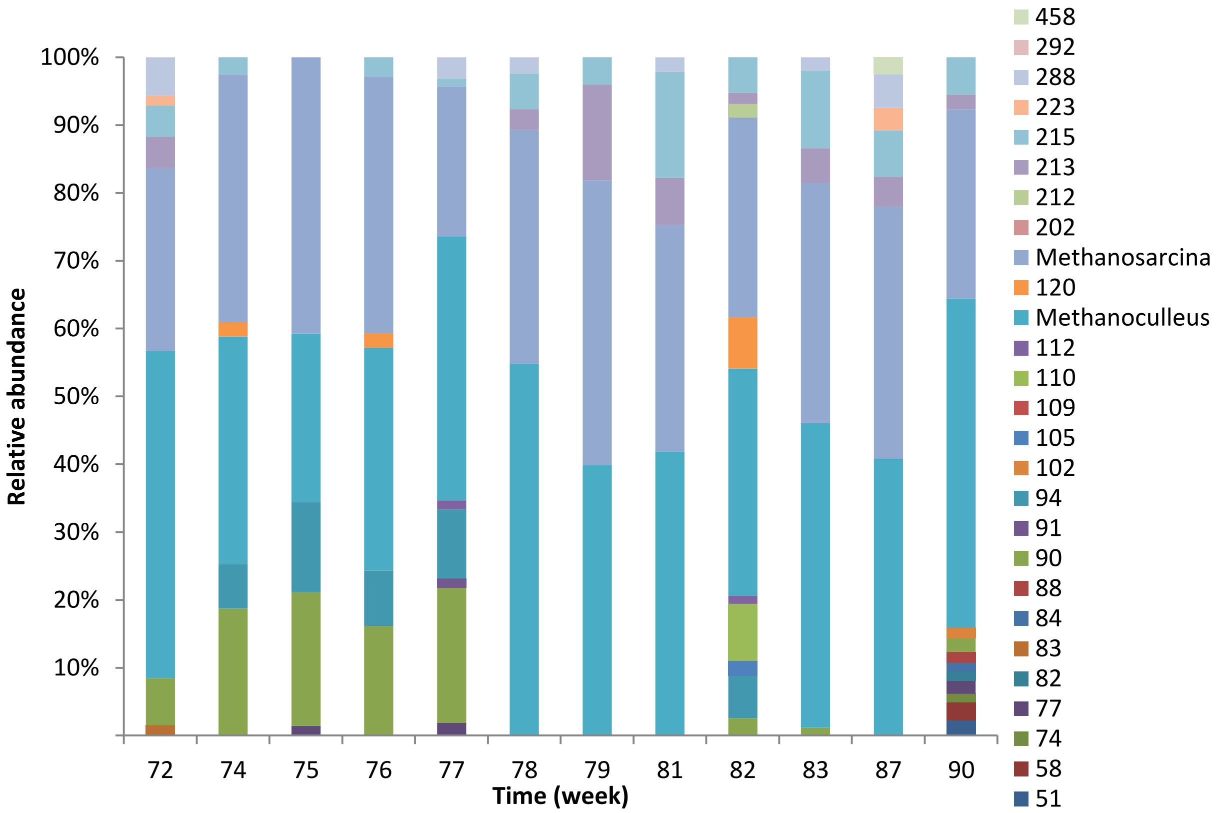

Figure S20. T-RFLP profiles of *mcr*A genes in samples from reactor R1.

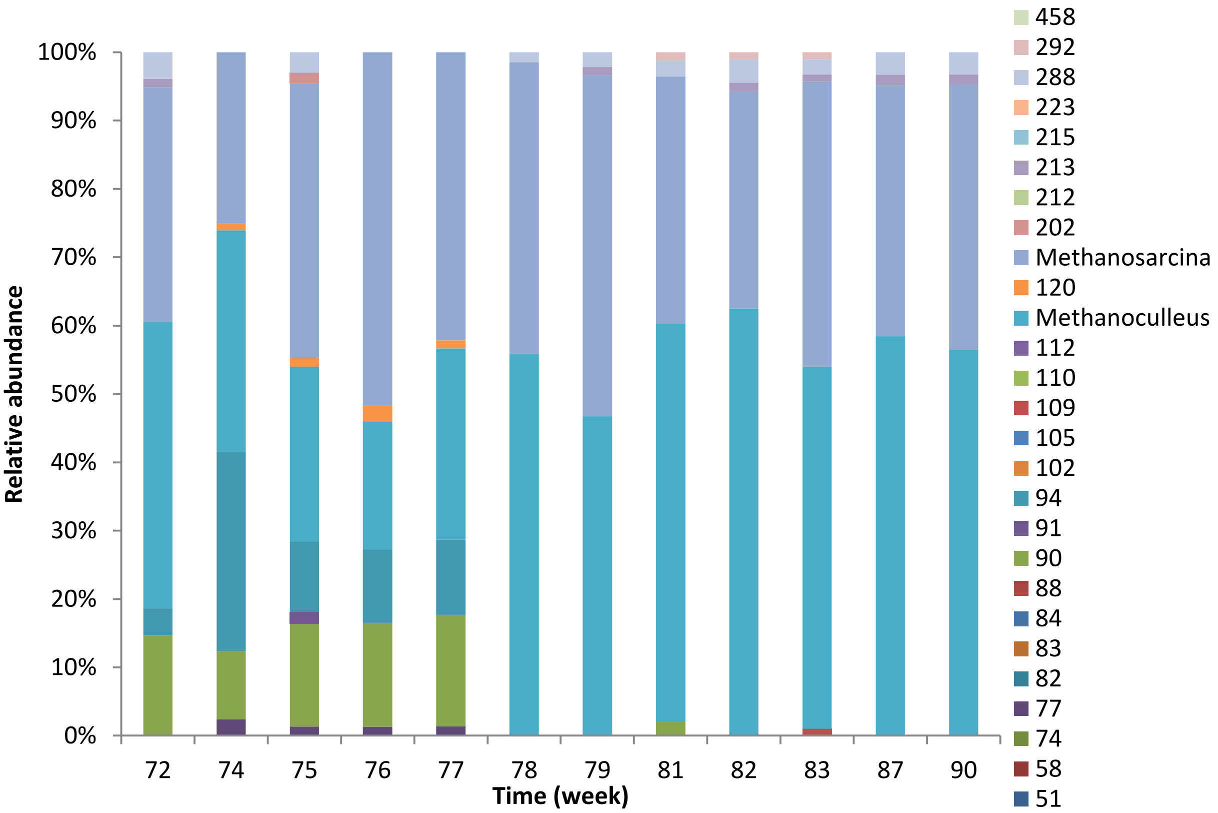

Figure S21. T-RFLP profiles of *mcr*A genes in samples from reactor R2.

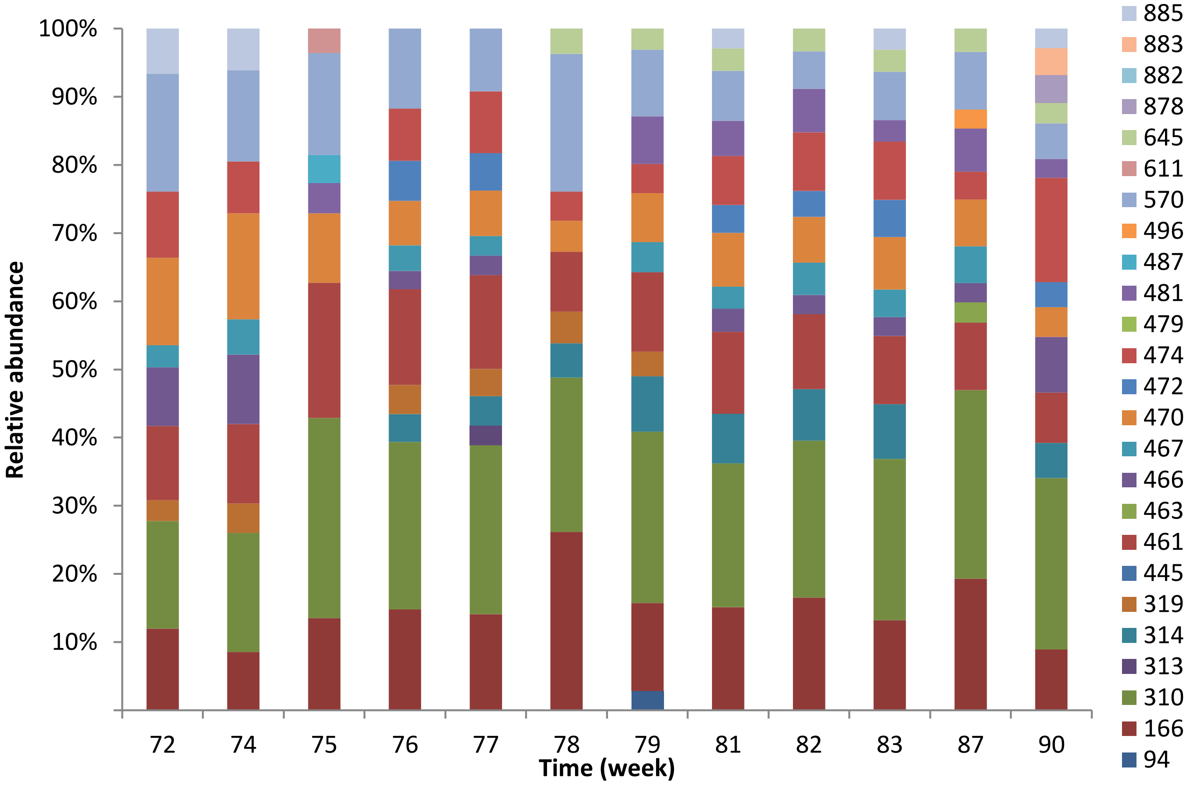

Figure S22. T-RFLP profiles of bacterial 16S rRNA genes in samples from reactor R1.

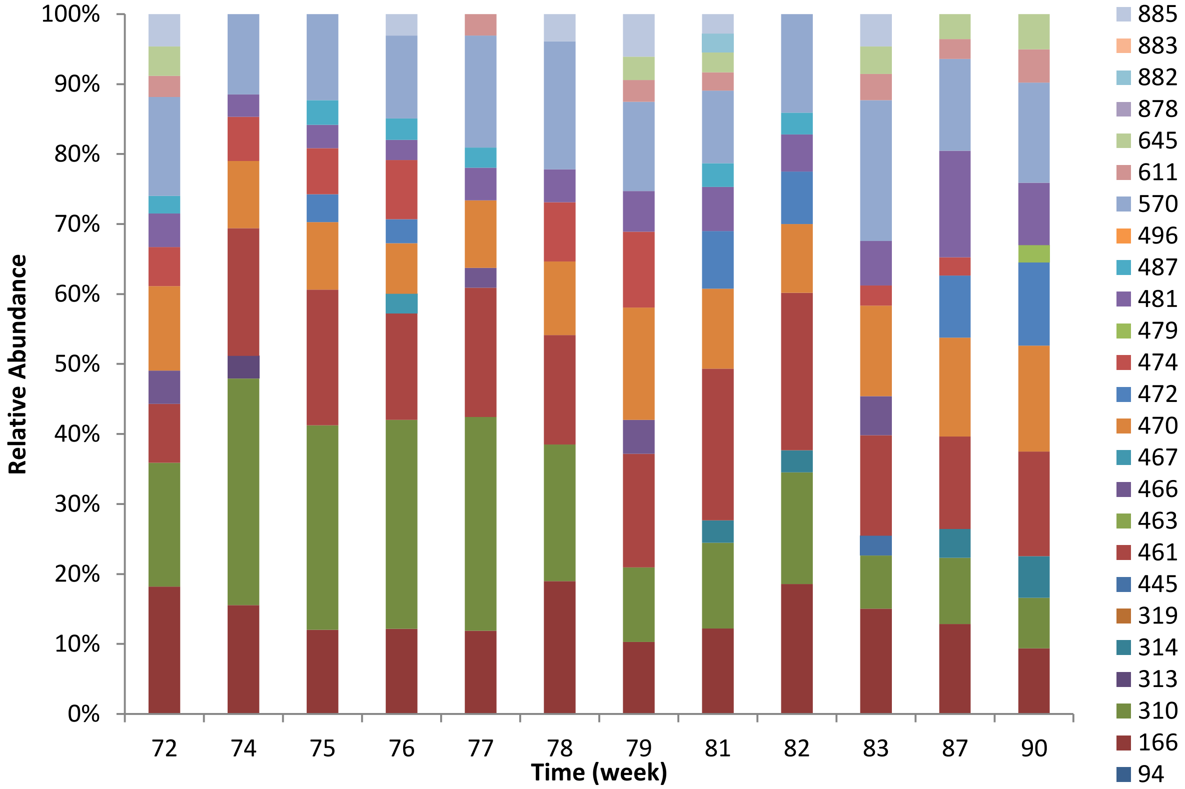

Figure S23. T-RFLP profiles of bacterial 16S rRNA genes in samples from reactor R2.

2.2 Supplementary Tables

Table S1. Number of reads, coverage (based on rarefaction analysis), observed richness (OTUs per sample), and diversity indices for 454 amplicon sequencing data

Sample	No. of reads	Coverage	Observed richness	Inverse Simpson index	Shannon index	Shannon index-based measure of evenness
**R1 Week 76**	12488	0.977979	662	30.198658	4.468264	0.687926
**R2 Week 76**	14414	0.977175	734	25.852281	4.525523	0.68584
**R1 Week 80**	15775	0.977813	778	31.766992	4.56121	0.685203
**R2 Week 80**	12488	0.978379	639	33.904502	4.508928	0.697987
